# Multivariate word properties in fluency tasks reveal markers of Alzheimer's dementia

**DOI:** 10.1002/alz.13472

**Published:** 2023-10-12

**Authors:** Franco J. Ferrante, Joaquín Migeot, Agustina Birba, Lucía Amoruso, Gonzalo Pérez, Eugenia Hesse, Enzo Tagliazucchi, Claudio Estienne, Cecilia Serrano, Andrea Slachevsky, Diana Matallana, Pablo Reyes, Agustín Ibáñez, Sol Fittipaldi, Cecilia Gonzalez Campo, Adolfo M. García

**Affiliations:** ^1^ Centro de Neurociencias Cognitivas Universidad de San Andrés Victoria Provincia de Buenos Aires Argentina; ^2^ Consejo Nacional de Investigaciones Científicas y Técnicas (CONICET) Ciudad Autónoma de Buenos Aires Argentina; ^3^ Facultad de Ingeniería Universidad de Buenos Aires (FIUBA) CABA Argentina; ^4^ Latin American Brain Health (BrainLat) Institute Universidad Adolfo Ibáñez Peñalolén Región Metropolitana Chile; ^5^ Center for Social and Cognitive Neuroscience (CSCN) School of Psychology Universidad Adolfo Ibáñez Las Condes Chile; ^6^ Instituto Universitario de Neurociencia Universidad de La Laguna La Laguna Tenerife España; ^7^ Cognitive Department of Psychology Universidad de La Laguna La Laguna Tenerife España; ^8^ Basque Center on Cognition Brain and Language (BCBL) San Sebastián Gipuzkoa España; ^9^ Ikerbasque Basque Foundation for Science Bilbao Spain; ^10^ Departamento de Matemática y Ciencias Universidad de San Andrés Victoria Provincia de Buenos Aires Argentina; ^11^ Departamento de Física Universidad de Buenos Aires and Instituto de Física de Buenos Aires (IFIBA‐CONICET) CABA Argentina; ^12^ Instituto de Ingeniería Biomédica Universidad de Buenos Aires Buenos Aires Argentina; ^13^ Unidad de Neurología Cognitiva Hospital César Milstein CABA Argentina; ^14^ Neuropsychology and Clinical Neuroscience Laboratory (LANNEC) Physiopathology Department ‐ ICBM Neurocience and East Neuroscience Departments Faculty of Medicine University of Chile Providencia Santiago Chile; ^15^ Geroscience Center for Brain Health and Metabolism (GERO) Faculty of Medicine University of Chile Providencia Santiago Chile; ^16^ Memory and Neuropsychiatric Clinic (CMYN) Neurology Department Hospital del Salvador and Faculty of Medicine University of Chile Providencia Santiago Chile; ^17^ Servicio de Neurología Departamento de Medicina Clínica Alemana‐Universidad del Desarrollo Las Condes Región Metropolitana Chile; ^18^ Instituto de Envejecimiento Department of Psychiatry School of Medicine Pontifical Xaverian University Bogotá Colombia; ^19^ Department of Mental Health Hospital Universitario Santa Fe de Bogotá Bogotá Colombia; ^20^ Centro de Memoria y Cognición Intellectus‐Hospital Universitario San Ignacio Bogotá Colombia; ^21^ Pontificia Universidad Javeriana Departments of Physiology Psychiatry and Aging Institute Bogotá Colombia; ^22^ Global Brain Health Institute, University of California San Francisco, San Francisco, California, USA Trinity College Dublin Dublin Ireland; ^23^ Departamento de Lingüística y Literatura Facultad de Humanidades Universidad de Santiago de Chile Estación Central Santiago Chile

**Keywords:** electroencephalography, machine learning, neurodegeneration, neuroimaging, semantic memory, word properties

## Abstract

**INTRODUCTION:**

Verbal fluency tasks are common in Alzheimer's disease (AD) assessments. Yet, standard valid response counts fail to reveal disease‐specific semantic memory patterns. Here, we leveraged automated word‐property analysis to capture neurocognitive markers of AD vis‐à‐vis behavioral variant frontotemporal dementia (bvFTD).

**METHODS:**

Patients and healthy controls completed two fluency tasks. We counted valid responses and computed each word's frequency, granularity, neighborhood, length, familiarity, and imageability. These features were used for group‐level discrimination, patient‐level identification, and correlations with executive and neural (magnetic resonanance imaging [MRI], functional MRI [fMRI], electroencephalography [EEG]) patterns.

**RESULTS:**

Valid responses revealed deficits in both disorders. Conversely, frequency, granularity, and neighborhood yielded robust group‐ and subject‐level discrimination only in AD, also predicting executive outcomes. Disease‐specific cortical thickness patterns were predicted by frequency in both disorders. Default‐mode and salience network hypoconnectivity, and EEG beta hypoconnectivity, were predicted by frequency and granularity only in AD.

**DISCUSSION:**

Word‐property analysis of fluency can boost AD characterization and diagnosis.

**Highlights:**

- We report novel word‐property analyses of verbal fluency in AD and bvFTD.
- Standard valid response counts captured deficits and brain patterns in both groups.
- Specific word properties (e.g., frequency, granularity) were altered only in AD.
- Such properties predicted cognitive and neural (MRI, fMRI, EEG) patterns in AD.
- Word‐property analysis of fluency can boost AD characterization and diagnosis.

## 1 BACKGROUND

As a complement to core learning and recall tests, Alzheimer's disease (AD) assessments typically include verbal fluency tasks.^1^ Participants have 1 min to produce words that begin with a given sound (phonemic fluency) or belong to a given category (semantic fluency).^2^ These tasks, especially in the semantic condition, reveal early and preclinical deficits^3^ which predict anatomo‐functional brain dysfunctions.^4^, ^5^ Moreover, they are brief, inexpensive, and massively available,^6^ highlighting their potential to reveal scalable AD markers.

Yet, performance is usually established by counting valid words—the sum of acceptable responses.^1^, ^7^ This standard scoring approach is blind to each word's linguistic features, precluding insights on which aspects of semantic memory are most affected.^1^ Also, it lacks diagnostic specificity, as it yields systematic deficits in other disorders, such as behavioral variant frontotemporal dementia (bvFTD).^8^ Indeed, beyond core sociobehavioral deficits, word retrieval is often compromised in bvFTD.^9^ Furthermore, standard scoring fails to capture disease‐differential neurocognitive disruptions. For example, valid responses correlate with executive outcomes^10^ and frontotemporal alterations^11^, ^12^, ^13^ in AD, but also in bvFTD.^14^ Finally, valid responses are derived subjectively from inconsistent criteria, compromising comparability and generalizability.^15^

These issues may be overcome through word‐property analysis. Each response can be decomposed into quantitative lexical variables, revealing word‐selection patterns during semantic memory search.^1^, ^16^ For instance, word frequency is abnormally high in AD.^1^ This variable predicts global Mini‐Mental Status Examination scores^17^ and might capture executive dysfunction, a predictor of dementia severity affected in most AD patients.^18^ Specific executive deficits (e.g., poor inhibition) may disfavor the retrieval of low‐frequency words (whose access increases inhibitory demands to suppress more accessible items).^19^ Moreover, frequency correlates with key neural vulnerabilities of AD,^20^, ^21^ including the volume of temporo‐parietal, fronto‐cingulate, and insular regions (measured via magnetic resonance imaging [MRI])^22^, ^23^ and connectivity along the default‐mode, salience, and executive networks (captured via fMRI).^23^

Also, persons with AD exhibit reduced semantic granularity, choosing conceptually coarse over specific words (*flower* instead of *rose*).^16^ This pattern could reflect memory and/or executive dysfunction (e.g., reduced abstraction capacity) as well as abnormal fMRI connectivity along temporal regions^21^ associated with conceptual precision.^24^ Furthermore, AD patients might favor words with many phonological neighbors—items with similar phoneme sequences, such as *house* relative to *mouse*.^25^ Indeed, as participants consider candidate words, those with several neighbors would be primed by activation of several shared phonemes.^25^ This would facilitate retrieval, biasing patients’ word choices given their difficulty navigating semantic memory.

Two further points merit consideration. First, specific word properties are associated with electroencephalography (EEG) connectivity in the beta (13–30 Hz) range,^26^ which is distinctly altered in AD.^27^ Second, semantic memory subdomains are more affected in AD than in bvFTD patients, whose deficits seem confined to specific categories, such as socio‐emotional concepts.^28^ Thus, word‐property analysis could yield syndrome‐differential anomalies across multiple modalities.

Here we report word‐property analyses of fluency outcomes in AD and bvFTD patients, compared with healthy controls (HCs). We counted valid responses and extracted distributional features of frequency, granularity, and phonological neighborhood, as well as complementary properties (length, familiarity, and imageability). In each patient sample, we examined which features yielded robust group‐level and subject‐level disease discrimination. Also, we examined whether sensitive word properties correlated with executive skills as well as structural (MRI), hemodynamic (functional MRI [fMRI]), and electrophysiological (EEG) brain alterations. We employed scalable automated methods and supervised machine learning —algorithms that capture complex patterns in multivariate datasets and weigh each feature's contribution to disease detection.^29^

**RESEARCH IN CONTEXT**

1. **Systematic review**: We reviewed verbal fluency studies in Alzheimer's disease (AD) and related disorders through PubMed and Google Scholar searches. The vast majority of studies restricted their analyses to valid response counts. Only a few considered the properties of words produced, and none integrated inferential and machine learning analyses of such features alongside correlations with multimodal neuroimaging measures. The word‐property approach was noted for its capacity to reveal specific aspects of semantic memory deterioration in AD. All relevant works were duly cited.
2. **Interpretation**: Our findings show that word‐property analysis can boost standard verbal fluency assessments by revealing markers of AD that are absent in behavioral variant frontotemporal dementia and which predict cognitive and multimodal (magnetic resonance imaging [MRI], functional MRI [fMRI], electroencephalography [EEG]) outcomes.
3. **Future directions**: This approach should be further validated in larger cohorts and additional disorders (to test their systematicity and specificity) and in longitudinal designs (to assess their usefulness to predict disease progression).

We predicted that both patient groups would produce fewer valid responses than HCs, but that only AD patients would be discriminated by responses’ frequency, granularity, and/or neighborhood. Moreover, we anticipated that such properties would correlate with executive outcomes and disease‐specific structural (MRI) and functional (fMRI, EEG) disruptions. In all cases, we examined whether predicted patterns differed between phonemic and semantic fluency.

## 2 METHODS

**FIGURE 1:**
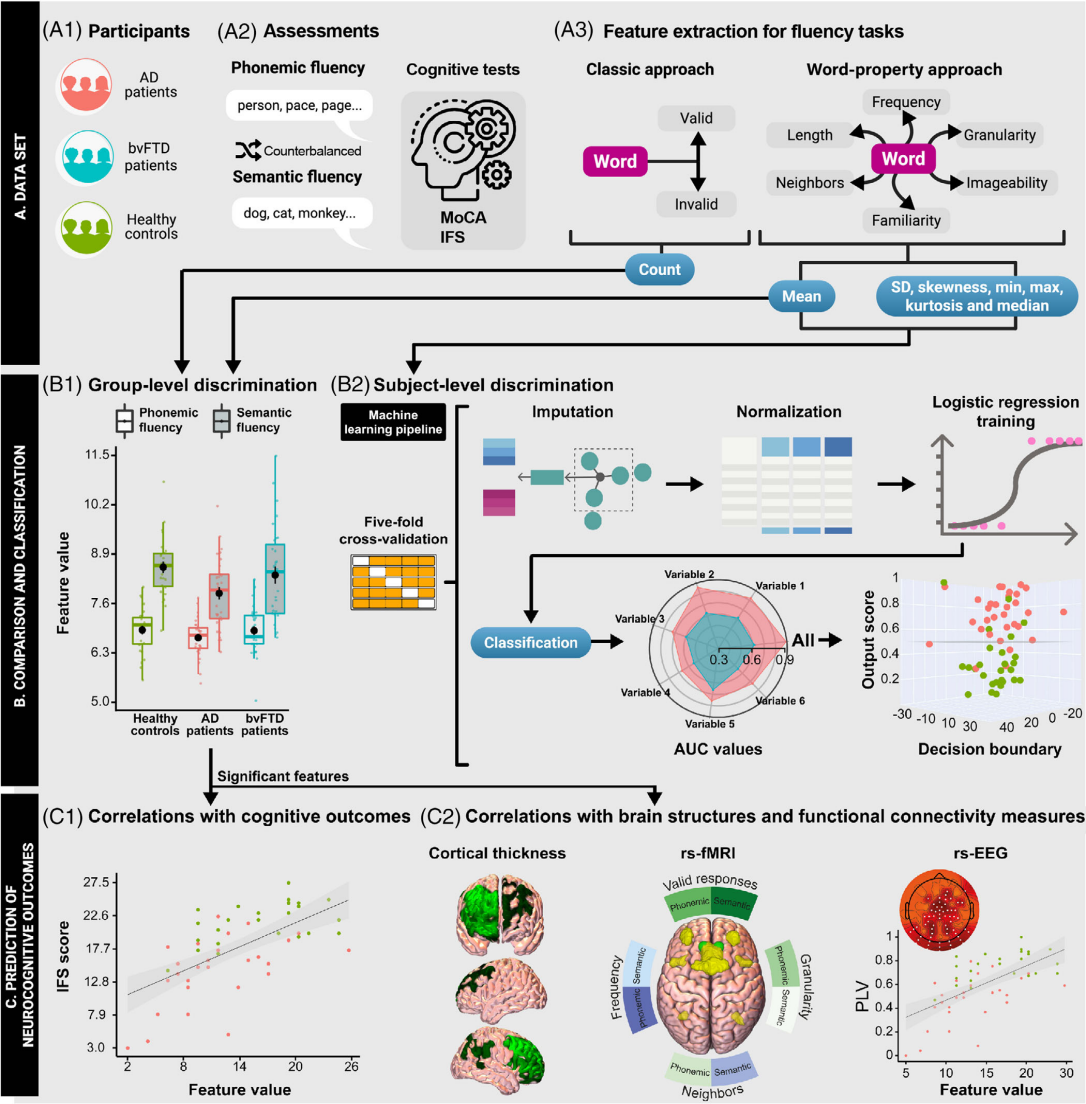
Experimental design. (A) Data set. (A1) We recruited persons with AD and bvFTD as well as healthy controls. (A2) Participants produced words starting with /p/ (phonemic fluency) or denoting animals (semantic fluency), and completed standard cognitive assessments (MoCA and IFS). (A3) Data for analysis was obtained through (i) the classic approach (number of valid responses) and (ii) our word‐property approach (where each word is decomposed into six variables, each characterized via seven distributional features). (B) Comparison and classification. (B1) Valid responses and the mean value of each word property were compared between each patient group and HCs via 2 × 2 mixed effects ANCOVAs (with the factors “group” and “task”, covarying for sex, age, and education) and Tukey's HSD test for post‐hoc comparisons. (B2) Logistic regressions were run for each word property and for their combination to classify between each patient group and HCs. (C) Prediction of neurocognitive outcomes. The mean of each word property yielding significant group differences was correlated with executive outcomes (C1) as well as with cortical thickness, resting‐state fMRI connectivity, and resting‐state EEG connectivity (C2). AD, Alzheimer's dementia; ANCOVA, analysis of covariance; AUC, area under receiver operating characteristic curve; bvFTD, behavioral variant frontotemporal dementia; fMRI, functional magnetic resonance imaging; HSD, honestly significant difference; HC, healthy control; IFS, INECO Frontal Screening; MoCA, Montreal Cognitive Assessment

The study's methods are summarized in Figure 1.

### 2.1 Participants

The study comprised 91 native Spanish speakers with normal or corrected‐to‐normal hearing and vision, namely: 32 persons with AD, 32 with bvFTD, and 27 HCs (Figure 1A1). This sample size reaches a power of 0.97 (Supplementary material 1). Participants were recruited in three centers from the Multi‐Partner Consortium to Expand Dementia Research in Latin America (ReDLat) following unified procedures.^30^ As in previous research,^31^ each patient group was matched with HCs for sex, handedness, age, and education (Table 1).

**TABLE 1:** Participants’ sociodemographic and cognitive profiles.

					Pairwise comparisons		
	Persons with AD *N* = 32	Persons with bvFTD *N* = 32	Healthy controls *N* = 27	Statistics (all groups)	Groups	Estimate	*p‐*Value
**Sociodemographic profiles**							
**Sex (F:M)**	18:14	17:15	19:8	–	AD‐HCs	0.72	0.40^a^
					bvFTD‐HCs	1.18	0.28^a^
**Handedness (L:R)**	3:23	0:25	1:19	–	AD‐HCs	0.06	0.80^a^
					bvFTD‐HCs	0.01	0.91^a^
**Years of age**	75.75 (5.63)	68.87 (7.81)	72.30 (7.34)	*F* = 7.70 *p <* 0.001^b^	AD‐HCs	1.90	0.11^c^
					bvFTD‐HCs	‐1.87	0.12^c^
**Years of education**	11.63 (4.20)	13.20 (5.01)	13.59 (3.78)	*F* = 1.72 *p =* 0.19^b^	AD‐HCs	‐1.72	0.16^c^
					bvFTD‐HCs	‐0.34	0.92^c^
**Cognitive profiles**							
**MoCA**	15.41 (4.98)	17.92 (7.61)	25.70 (3.27)	*F* = 25.92 *p <* 0.001^b^	AD‐HCs	‐6.95	< 0.001^c^
					bvFTD‐HCs	‐5.12	< 0.001^c^
**IFS**	14.41 (4.96)	15.66 (7.37)	21.43 (3.06)	*F* = 13.38 *p <* 0.001^b^	AD‐HCs	‐4.90	< 0.001^c^
					bvFTD‐HCs	‐3.88	0.002^c^

*Note*: Data presented as mean (SD), except for sex and handedness. (a) *p*‐Values calculated via chi‐squared test (χ2). (b) *p*‐Values calculated via independent measures ANCOVA; (c) *p*‐Values calculated via Dunnett's test.

Abbreviations: AD, Alzheimer's dementia; bvFTD, behavioral variant frontotemporal dementia; HC, healthy control; IFS, INECO Frontal Screening; MoCA, Montreal Cognitive Assessment.

Persons with AD were diagnosed by expert neurologists following clinical criteria from the National Institute of Neurological and Communicative Diseases and Stroke as well as the Alzheimer's dementia and Related Disorders Association.^32^, ^33^ They lacked functional autonomy and exhibited predominant temporo‐parietal atrophy (Supplementary material 2). Persons with bvFTD were diagnosed following current criteria.^34^ They all exhibited socio‐behavioral impairments as defined by caregivers^35^ and predominantly frontotemporal atrophy, involving insular and cingulate cortices (Supplementary material 2). Results from the Montreal Cognitive Assessment (MoCA)^2^ and the INECO Frontal Screening (IFS) battery (Supplementary material 3)^36^ revealed that both patient groups had moderate cognitive impairment and executive dysfunction (Table 1). Diagnoses were supported by extensive neurological, neuropsychiatric, and neuropsychological examinations.^28^, ^35^ No patient reported a history of other neurological disorders, psychiatric conditions, primary language deficits, or substance abuse. HCs were recruited through an outreach program and invitations to patients’ eligible caregivers. These participants had no background of neuropsychiatric disease or alcohol/drug abuse and, based on a neurological interview, they were confirmed to be functionally autonomous and cognitively preserved, with MoCA scores above the local cutoff of 21.^37^ Across the three groups, all participants completed the neuropsychological, neuroimaging, and EEG assessments in a mean span of less than a month.

### 2.2 Fluency tasks

All participants completed phonemic and semantic fluency tasks (Figure 1‐A2), requiring them to utter as many words as they could say starting with the phoneme /p/ and belonging to the category “animals”, respectively. These were administered in counterbalanced fashion by a certified neuropsychologist, in a silent testing room, always before the MoCA and the IFS battery (no further tests were included in these sessions). Following standard procedures,^2^ participants were given 1 min per fluency task and instructed not to produce proper names, numbers, repetitions, words from the same family, or morphological variations of the same word. Instructions were provided orally, including examples of invalid responses. Responses were audio‐recorded, transcribed by one examiner, and then checked by another. The few cases of discrepancy were settled by a third examiner. Unintelligible words were discarded.

### 2.3 Standard approach

Based on the standard approach, performance was measured as the number of valid responses (Figure 1‐A3, left inset). Words that did not comply with the instructions were framed as invalid. Individual scores for each task were computed as the total number of valid responses.

### 2.4 Word‐property approach

For the word‐property approach, each response was analyzed in terms of six variables (Figure 1‐A3, right inset). We used the EsPal database^38^ to derive each word's frequency (logarithmic frequency per million), phonological neighborhood (number of words obtained upon substituting, adding, or omitting a phoneme), *length* (number of phonemes), familiarity (from 1: not familiar to 7: highly familiar), and imageability (from 1: not imageable to 7: highly imageable). EsPal is the largest psycholinguistic database for Spanish, based on information from 300 million written tokens for corpus‐based variables (e.g., frequency, phonological neighborhood, length) and normative data from native speakers for subjective variables (e.g., familiarity, imageability). Specifically, we used Python to create an ad‐hoc script that automatically accessed the EsPal website, uploaded a file containing each word produced, and retrieved the corresponding values. Then, to calculate each word's *granularity*, we used Python's NLTK library^39^ to access WordNet, a hierarchical graph of nodes leading from the highest hypernym (“entity”) to progressively more specific concepts (e.g., “animal”, “dog”, “bulldog”).^16^ Granularity is defined as the number of nodes between a word and its related “entity” (e.g., bin‐3 words are closer to “entity” than bin‐10 words, the former indicating more general concepts).

Words with no value in any given property were ignored. Comparisons of such missing data yielded non‐significant effects of group and group‐by‐task interactions for every property in each group pair (all *p‐v*alues > 0.06), corroborating data comparability across groups (Supplementary material 4). Finally, to maximally exploit our multivariate approach, as in previous research,^40^ each property was analyzed in terms of seven distributional features, namely: mean, median, standard deviation, minimum, maximum, skewness, and kurtosis (Figure 1‐A3, right inset).

### 2.5 Behavioral data analysis

#### 2.5.1 Group‐level discrimination

The features described above were statistically compared between (a) persons with AD and HCs, and (b) persons with bvFTD and HCs (Figure 1‐B1). In each case, valid responses and the mean value of each word property (across all valid and invalid responses) were compared between groups via mixed effects analyses of covariance (ANCOVAs), with “group” as between‐subjects factor, “task” as within‐subject factor, and sex, age, and years of education as covariates. Post‐hoc comparisons for significant interaction effects were performed via Tukey's honestly significant difference (HSD) tests. Alpha levels were set at  *<* 0.05. Effect sizes were calculated through partial eta squared (η_p_^2^) tests for main and interaction effects, and through Cohen's *d* for post‐hoc pairwise comparisons. To ensure that results were not driven by specific data trimming procedures, we replicated all analyses upon excluding (i) invalid responses (as defined above) and (ii) outlier responses (beyond 3 *SD*s from the mean of the participant's group). ANCOVAs and post‐hoc tests were performed on Pingouin v.0.5.1,^41^ and effect sizes calculations on G^*^Power v.3.1.^42^ Boxplots were created on R's ggplot2 library.

#### 2.5.2 Subject‐level discrimination

To explore the usefulness of the word‐property approach for probabilistic subject‐level discrimination, we ran machine learning analyses to classify between each patient group and HCs (Figure 1‐B2). For each pair, we ran a separate classifier considering the distributional features of each word property and then another one combining all features (*n* = 42), in a fully multivariate setting. The former strategy revealed the individual contribution of each property upon including all of its statistical features, whereas the latter provided an integrative classification score in a fully multivariate setting. Importantly, classifiers were fed exclusively with word‐property features, without any accompanying clinical, neuropsychological, or neuroimaging measure. In each case, data were randomly divided into five folds for stratified cross‐validation, preserving the proportion of labels per group,^43^ with four folds used for training and one for testing. Values for each feature were normalized using the min‐max method^43^ and missing data per participant were imputed using *K*‐Nearest Neighbors using uniform weights and *K* = 5. We used a logistic regression classifier with default hyperparameter values, a robust method capturing neuropsychological and psycholinguistic patterns in dementia^44^ (Supplementary material 5). This method models probabilities based on a logistic function to smoothly limit the output score from 0 to 1, as recommended for feature‐to‐sample ratios similar to ours.^45^ Classifier performance is reported as mean and *SD* obtained upon 1000 iterations with different random partitions of the data. Over the course of the 1000 iterations, the absolute values of each feature coefficient were calculated for a feature importance analysis. All analyses were performed on Python 3.9 and the Scikit‐learn (https://scikit‐learn.org/) package. Radar plots and boundary decision plots were created with the Plotly library on R and Python, respectively.

#### 2.5.3 Correlations with cognitive outcomes

To estimate whether the standard and the word‐property approaches could capture disease‐specific executive outcomes, the participants’ mean value in each variable yielding significant group effects was correlated with their global IFS scores (Figure 1‐C1). To increase variance and statistical power, as in previous neurocognitive studies,^46^ these analyses were conducted collapsing each patient group with HCs. Correlations were performed with Spearman's indices, correcting for the number of correlations per analysis via the false discovery rate (FDR) method. Correlation analyses were performed on R (v.1.4.1717).

### 2.6 Analysis of neural patterns and brain‐behavior correlations

#### 2.6.1 Neuroimaging

##### Data acquisition

MRI and fMRI recordings were obtained from 20 persons with AD and 18 with bvFTD, all matched with 20 HCs for sex, handedness, age, and education (Supplementary material 6). Recordings were performed in three scanners, with minimally varying parameters (Supplementary material 7). Acquisition center was introduced as a covariate in all neuroimaging analyses. During the fMRI session, participants were asked to not to think about anything in particular and to remain still, awake, and with eyes closed.

##### Structural imaging: Preprocessing and analysis

Participants’ cortical thickness was estimated via surface‐based morphometry. Preprocessing and analysis were performed with CAT12 (http://www.neuro.uni‐jena.de/cat), based on SPM12 (https://www.fil.ion.ucl.ac.uk/spm/) on MATLAB R2021a. Preprocessing steps followed CAT12 guidelines (http://www.neuro.uni‐jena.de/cat12/CAT12‐Manual.pdf). First, images were segmented and normalized based on a surface and thickness estimation. Second, cortical thickness images were resampled and smoothed employing a 12‐mm kernel. Third, hemisphere images were merged to obtain a single cortical thickness image per subject. Finally, sample homogeneity and orthogonality were quality‐checked.

Patients’ atrophy was estimated by comparing their cortical thickness with that of HCs via ANCOVAs, controlling for acquisition center. Then, with standard SPM12 module calling CAT12 functions, multiple regressions were run to test for correlations between each significant verbal fluency measure and cortical thickness, again controlling for acquisition center (Figure 1‐C2, left inset). These analyses were performed collapsing each patient group with HCs to increase sample size, data variance, and statistical power, as per.^31^ Both analyses were corrected by the threshold‐free cluster enhancement (TFCE) method,^47^ running on the TFCE toolbox (http://www.neuro.uni‐jena.de/tfce), which is an extension of SPM12. By taking raw statistics from the images and producing a transformed output image in which the voxel‐wise values reflect the number of cluster‐like features, this method circumvents the arbitrary selection of a hard threshold for cluster estimation. Also, as it relies on permutation testing, TFCE is robust against spurious results due to multiple comparisons.^47^ We performed 5000 permutations. The alpha level was set at *p <* 0.05, FDR‐corrected.

##### Functional imaging: Preprocessing and analysis

Preprocessing was performed on the Data Processing Assistant for Resting‐State fMRI (DPARSF v.6.1)^48^ software, calling Statistical Parametrical Mapping 12 (SPM12) and resting‐state fMRI Data Analysis Toolkit (REST v.1.8)^49^ functions. To ensure that magnetization achieved a steady state, we discarded the first five volumes of each recording before preprocessing. First, images were slice‐time corrected, referenced to the middle slice of each volume, and realigned to the first scan to correct for head movements. Second, images were normalized to the MNI space employing the Echo‐Planar Imaging template provided by SPM. Third, bandpass filtering (0.01–0.1 Hz) and smoothing (8‐mm full‐width‐at‐half‐maximum isotropic Gaussian kernel) were applied. To reduce the confounding effects of physiological and motion artifacts, six motion parameters, white matter, cerebrospinal fluid, and global signals were regressed. White matter and cerebrospinal fluid masks were derived from the tissue segmentation of the subjects’ T1 recording in native space. Finally, motion parameters were obtained from the realignment step and matched between each patient group and HCs (Supplementary material 8).

As in previous works,^46^ bilateral seeds were established to evaluate the functional connectivity of the default‐mode network (seeds: posterior cingulate cortices), the salience network (seeds: dorsal anterior cingulate cortices), the executive network (seeds: superior frontal gyri), and the semantic network (seeds: ventral anterior temporal lobes), all relative to the rest of the brain. Connectivity maps were averaged for each network to obtain connectivity strength values, based on the weighted Symbolic Dependence Metric (wSDM).^50^ This validated, non‐linear metric captures the local and global temporal features of the blood‐oxygen‐level‐dependent (BOLD) signal by weighing a robust copula‐based dependence measure by symbolic similarity.^50^ For details, see Supplementary material 9.

The functional connectivity patterns of each patient group were estimated by comparisons with HCs via ANCOVAs, controlling for acquisition center. Then, associations between each significant verbal fluency measure and each network yielding connectivity differences were examined via partial correlation analyses, again controlling for acquisition center (Figure 1‐C2, middle inset). As for cortical thickness, analyses were performed collapsing each patient group with controls to increase sample size, data variance, and statistical power.^31^ Pearson's or Spearman's partial correlation tests were applied depending on the variables’ normal or non‐normal distribution, respectively, as shown by Shapiro‐Wilk test results. All comparisons and correlations were FDR‐corrected at *p* <  0.05.

#### 2.6.2 Exploratory EEG analyses

##### Data acquisition

Sixteen persons with AD, 11 with bvFTD, and 14 HCs completed a 10‐min resting‐state EEG protocol.^28^ These sub‐samples remained sociodemographically matched (Supplementary material 10). Participants were instructed not to think about anything in particular while keeping still, awake, and with eyes closed. High‐density EEG recordings were acquired with a Biosemi‐active‐two 128‐channel system (Amsterdam, NLD) at a sampling rate of 1024 Hz.

##### Preprocessing and analysis

Signals were band‐pass filtered offline between 0.03 and 100 Hz. A digital bandpass filter between 0.5 and 45 Hz was further applied to remove unwanted frequency components. During recording, the reference was set as default to link mastoids and re‐referenced offline to the average of all electrodes.^51^ Bad channels were replaced via statistically weighted spherical interpolation method (based on all sensors).^52^ All EEG signal processing steps were implemented on MATLAB software (vR2016a) through the EEGLAB toolbox (v14.1.2).^53^ Signals contaminated with eye movements or blink artifacts were corrected with independent component analysis^54^ and with a visual inspection protocol. Clean resting‐state recordings were then divided into 1000‐ms segments and used for functional connectivity analysis. Importantly, pairwise comparisons on the number of valid segments did not differ significantly (all *p‐v*alues > 0.14) between each patient group (persons with AD: *M* = 418, *SD* = 149; persons with bvFTD: *M* = 368, *SD* = 186) and HCs (*M* = 458, *SD* = 116).

Pairwise EEG functional connectivity values for each electrode site were quantified with the phase‐locking value (PLV) method,^55^ which measures linear interactions between oscillatory signals. PLV is a measure of phase synchronization that captures the instantaneous phase difference of two brain signals on the assumption that connected areas generate signals whose instantaneous phases evolve together. More specifically, PLV evaluates the spread of the distribution of phase differences, and the connectivity estimation is linked to this spread. The narrower the distribution of the phase difference, the higher the PLV value—which ranges between 0 (i.e., the phase of the two EEG signals is not synchronized) and 1 (i.e., the phase of the two EEG signals is perfectly synchronized). Functional connectivity value was computed at the beta (13–30 Hz) frequency band. To identify disease‐specific connectivity patterns, we performed non‐parametric cluster‐based permutations for independent samples,^56^ comparing healthy controls to each patient group. As in previous studies,^28^ cluster‐level statistics were based on the number of connections in the largest cluster obtained in each permutation. To identify connections that could form significant clusters we performed two‐tailed t‐tests at *p*_con_ < 0.05. Clusters with *P*_clus_ < 0.05 were considered significant. We estimated the *p‐v*alue of each cluster as the proportion of 1000 random permutations of the connectivity matrices that yielded a cluster‐level statistic greater than that of the corresponding cluster in the observed data.

Finally, given that variables were normally distributed and that equipment and acquisition parameters were identical across centers, we tested bivariate Pearson's correlations between the significant clusters’ mean connectivity and values in each word variable yielding between‐group differences in ANCOVAs (Figure 1‐C2, right inset). As in previous language research on neurodegeneration,^28^ we used FDR to correct for the number of correlations per analysis.

## 3 RESULTS

### 3.1 Group‐level discrimination

**FIGURE 2:**
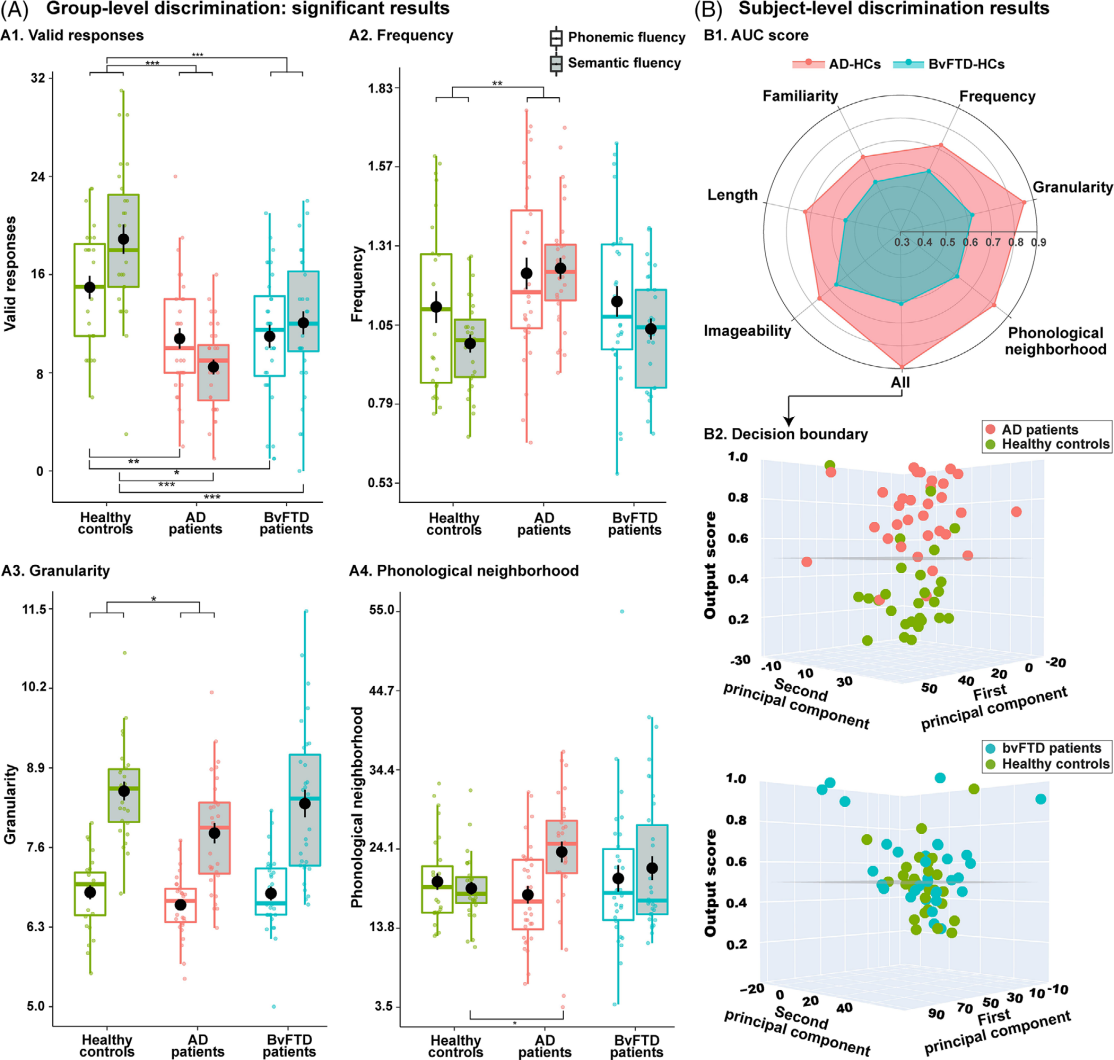
Main results. (A) Significant group‐level results, based on mixed‐effects ANCOVAs, with the factors group and task, covarying for sex, age, and education. Top‐brackets indicate significant main effects of group, and bottom‐brackets indicate significant pairwise differences in interaction effects (^***^ *p* < 0.001; ^**^ *p* < 0.01; ^*^ *p* < 0.05). (A1) All patient groups produced fewer valid responses than HCs. (A2‐4). Significant differences in word properties were found only for persons with AD, typified by higher frequency and lower granularity than HCs across tasks, as well as higher phonological neighborhood in the semantic task. No word property yielded significant differences between persons with bvFTD relative to HCs. (B) Logistic regressions were used to classify between each patient group and HCs based on distributional information from each word property in both tasks, and then for all properties combined. (B1) The radar plot showed maximal discrimination between AD and HCs based on features from all properties, with good results for each property in isolation. Classification was substantially lower between persons with bvFTD and HCs. (B2) Output scores based on all features together revealed good detection of persons with AD and HCs using a decision boundary of 0.5, with marked confusion for the other group pair. Final output score per subject was obtained after averaging it over the 1000 iterations. AD, Alzheimer's dementia; ANCOVA, analysis of covariance; AUC, area under receiver operating characteristic curve; bvFTD, behavioral variant frontotemporal dementia; HC, healthy control

The standard approach revealed significantly fewer valid responses in each patient group compared with HCs [AD: *F*(1,57) = 55.21, *p <* 0.01, η_p_^2^ = 0.42; bvFTD: *F*(1.57) = 22.66, *p <* 0.01, η_p_^2^ = 0.24]. These effects were qualified by an interaction with task in persons with AD (*MSE* = 13.99, *df* = 57) and with bvFTD (*MSE* = 9.97, *df* = 57), both outperformed by HCs on each task separately (all *p‐v*alues ≤ 0.03) (Figure 2‐A1). For full results, see Supplementary material 11.

Conversely, the word‐property approach revealed differences in persons with AD only. Main group effects were observed for frequency [*F*(1.57) = 9.46, *p <* 0.01, η_p_^2^ = 0.12] and granularity [*F*(1.57) = 4.63, *p =* 0.03, η_p_^2^ = 0.05], without accompanying interaction effects (Figure 2‐A2, 2‐A3). Also, analysis of phonological neighborhood revealed a group‐by‐task interaction (*MSE* = 28.36, *df* = 57), with post hoc comparisons (Figure 2‐A4) showing higher values for persons with AD than HCs on the semantic task (*p =* 0.03, *d* = 0.74), and for persons with AD on the semantic than on the phonemic task (*p <* 0.01, *d* = 0.75) —an effect that was not mirrored in HCs (*p =* 0.97, *d* = 0.15). The remaining properties yielded non‐significant main effects of group (all *p‐v*alues > 0.07) and interaction effects (all *p‐v*alues > 0.25). Comparisons of persons with bvFTD relative to HCs did not yield significant group or interaction effects in any property (all *p‐v*alues > 0.06). For full results, see Supplementary material 11. Importantly, all these significant and non‐significant effects remained similar upon removing invalid responses and outliers (Supplementary material 12).

### 3.2 Subject‐level discrimination

Maximal classification between persons with AD and HCs was obtained upon considering distributional information from all word properties (area under the curve [AUC] = 0.89 ± 0.09), with frequency, granularity, and phonological neighborhood figuring prominently among the top discriminating features. Compatibly, analyses of individual properties revealed high discrimination based on granularity (AUC = 0.86 ± 0.10) and phonological neighborhood (AUC = 0.82 ± 0.11), followed by frequency, imageability, and length (all AUCs > 0.70). The same multivariate classifiers yielded lower discrimination between persons with bvFTD and HCs (AUC = 0.62 ± 0.15). AUC scores are shown in Figure 2‐B1, and decision boundary based on features from all properties combined are shown in Figure 2‐B2. For details and top features, see Supplementary material 13. An exploratory classification between AD and bvFTD patients yielded above‐chance results (AUC = 0.63 ± 0.13, accuracy = 0.63 ± 0.13).

### 3.3 Correlations between fluency measures and executive outcomes

**FIGURE 3:**
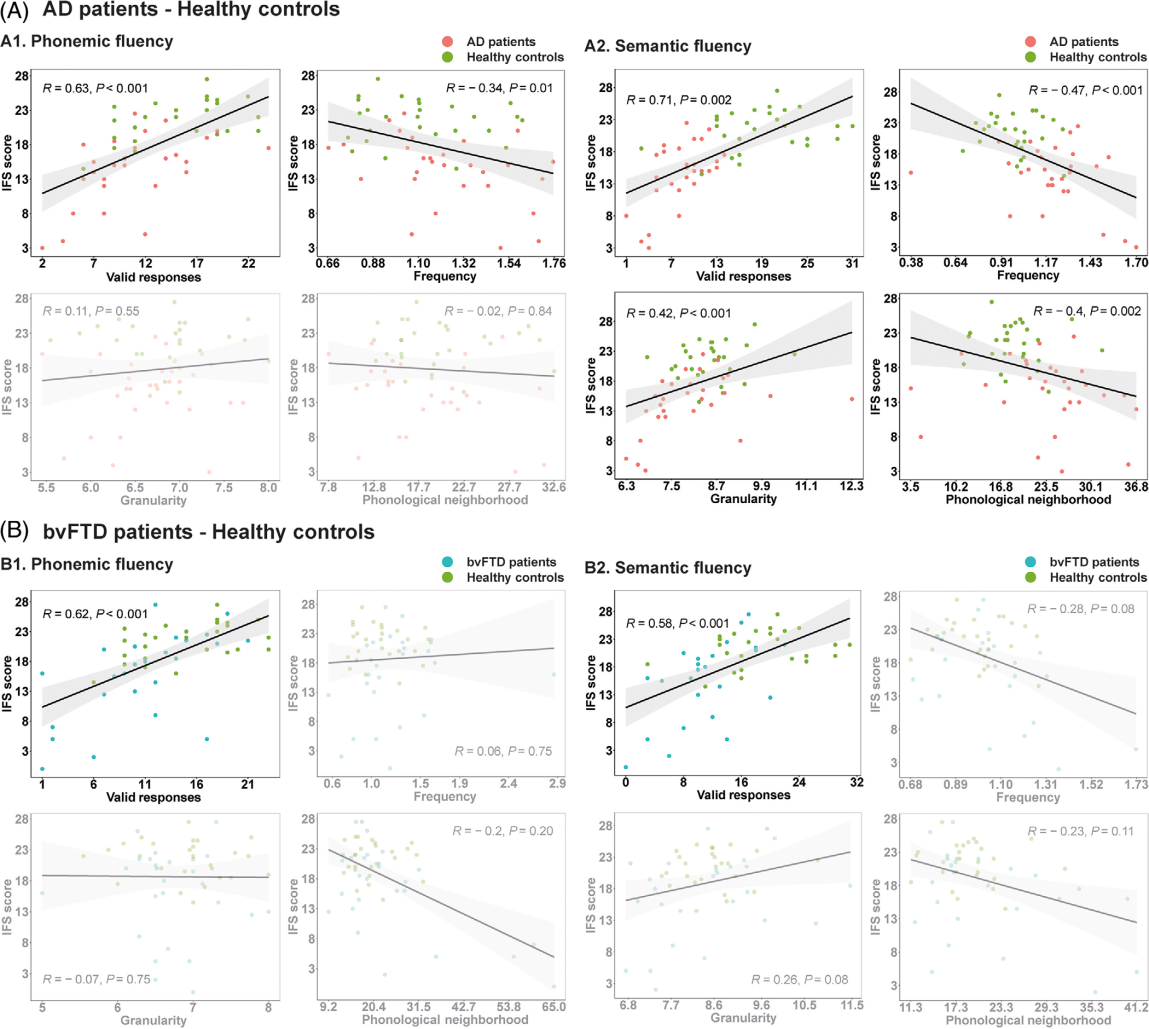
Spearman correlations between significant group‐level features and cognitive outcomes. (A) Correlations for the AD‐HC analysis based on phonemic (A1) and semantic (A2) fluency outcomes revealed that IFS scores were predicted by valid responses as well as significant word‐property variables. (B) Correlations for the bvFTD‐HC analysis based on phonemic (B1) and semantic (B2) fluency outcomes revealed that IFS scores were predicted by valid responses only. Analyses used the false discovery rate (FDR) method to control for multiple correlations per analysis. Non‐significant results are shown with a gray mask. AD, Alzheimer's disease; bvFTD, behavioral variant frontotemporal dementia; HC, healthy control; IFS, INECO Frontal Screening

Valid responses on both the phonemic and the semantic conditions correlated with executive scores (IFS) scores in both the AD‐HC and the bvFTD‐HC analyses. The three word properties yielding significant ANCOVA results correlated with IFS scores in the AD‐HC analysis, with higher *R*‐values in the semantic task. These variables did not correlate with IFS scores in the bvFTD group. See Figure 3 for details.

### 3.4 Neuroimaging results

#### 3.4.1 Correlations between fluency variables and cortical thickness

**FIGURE 4:**
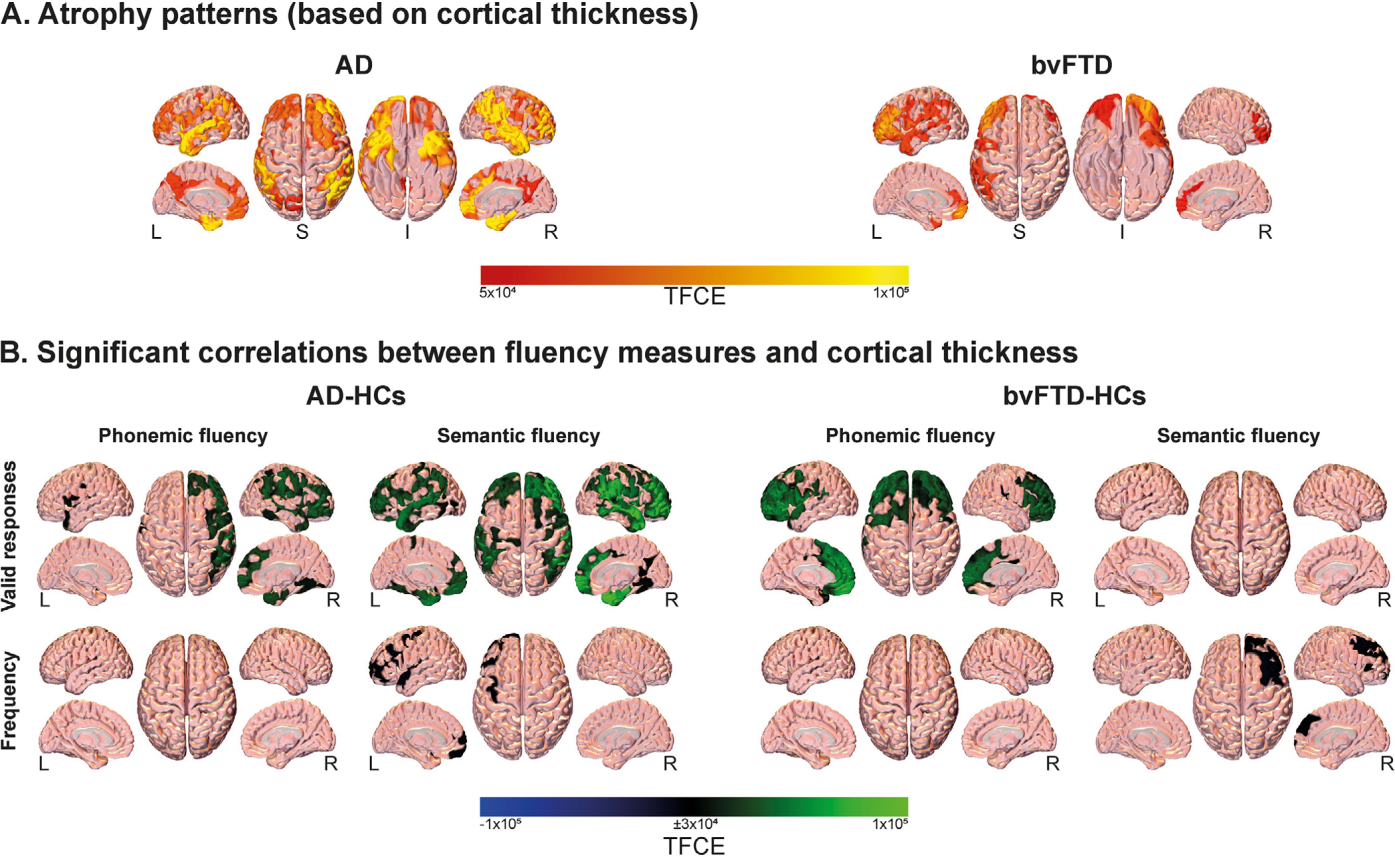
Surface‐based morphometry results. (A) Atrophy of persons with AD and bvFTD, relative to healthy controls. Reduced cortical thickness was observed predominantly in temporo‐parietal areas for AD and frontotemporal regions for bvFTD. (B) Associations between significant fluency variables and whole‐brain cortical thickness. Valid responses in the phonemic and semantic tasks were positively correlated with cortical thickness in various brain regions in the AD‐HC and bvFTD‐HC groups. In the AD‐HC group, valid responses in the phonemic task were positively correlated with cortical thickness in the left putamen, middle and posterior cingulate gyri, calcarine fissure, lingual gyrus, and right rolandic operculum, while valid responses in the semantic task were positively correlated with thickness in the left middle temporal gyrus and the right superior temporal and middle cingulate gyri. In the bvFTD‐HC group, valid responses in the phonemic task were positively correlated with thickness in the left anterior cingulate, precentral gyrus, middle occipital lobe, and calcarine fissure, as well as the right middle superior frontal gyrus, the right parahippocampus, and the bilateral posterior cingulate gyrus and precuneus. Frequency in the semantic task was linked to cortical thickness in both AD‐HC and the bvFTD‐HC groups, involving temporo‐parietal and cingulate cortices in the AD‐HC analysis, and frontotemporal and cingulate cortices in the bvFTD‐HC analysis. AD, Alzheimer's dementia; bvFTD, behavioral variant frontotemporal dementia; HC, healthy control; I, inferior; L, left; R, right; S, superior; TFCE, threshold‐free cluster enhancement

Distinct atrophy patterns were observed in each group, affecting mainly temporal, parietal, and frontal regions in AD, and fronto‐insulo‐temporal regions in bvFTD (Figure 4A). In the AD‐HC analysis (Figure 4B, left inset), valid responses in the phonemic task positively correlated with cortical thickness of the left putamen, middle and posterior cingulate gyri, calcarine fissure, and lingual gyrus, as well as the right rolandic operculum (*P*_FDR_ < 0.05). Regarding the semantic task, valid responses positively correlated with the thickness of the left middle temporal gyrus and the right superior temporal and middle cingulate gyri (*P*_FDR_ < 0.05). As regards word‐property variables, frequency in the semantic task negatively correlated with the thickness of the left superior temporal pole as well as the right supramarginal and middle cingulate gyri (*P*_FDR_ < 0.05). No other significant correlations were observed. For details, see Supplementary material 14.

In the bvFTD‐HC analysis (Figure 4B, right inset), valid responses in the phonemic task positively correlated with the thickness of the left anterior cingulate, precentral gyrus, middle occipital lobe, and calcarine fissure, as well as the right middle superior frontal gyrus, the right parahippocampus, and the bilateral posterior cingulate gyrus and precuneus (*P*_FDR_ < 0.05). As regards word properties, frequency in the semantic task negatively correlated with the thickness of the left middle superior frontal as well as the posterior, middle, and anterior cingulate gyri; the left middle and superior occipital lobes; the right orbital middle frontal as well as the inferior, middle, and superior temporal gyri; the right amygdala, parahippocampus, and putamen; and the bilateral middle frontal gyrus, cuneus, and supramarginal gyrus (*P*_FDR_ < 0.05). No other significant correlations were observed. For details, see Supplementary material 14.

#### 3.4.2 FMRI connectivity differences and correlations with fluency measures

Relative to HCs, both patient groups presented hypoconnectivity in the default‐mode, salience, and executive networks (*P*_FDR_ < 0.05). No network yielded hyperconnectivity in any patient group (all *P*_FDR_ values > 0.05). No other significant pairwise comparisons emerged for any other network (all *P*_FDR_ values > 0.05).

**FIGURE 5:**
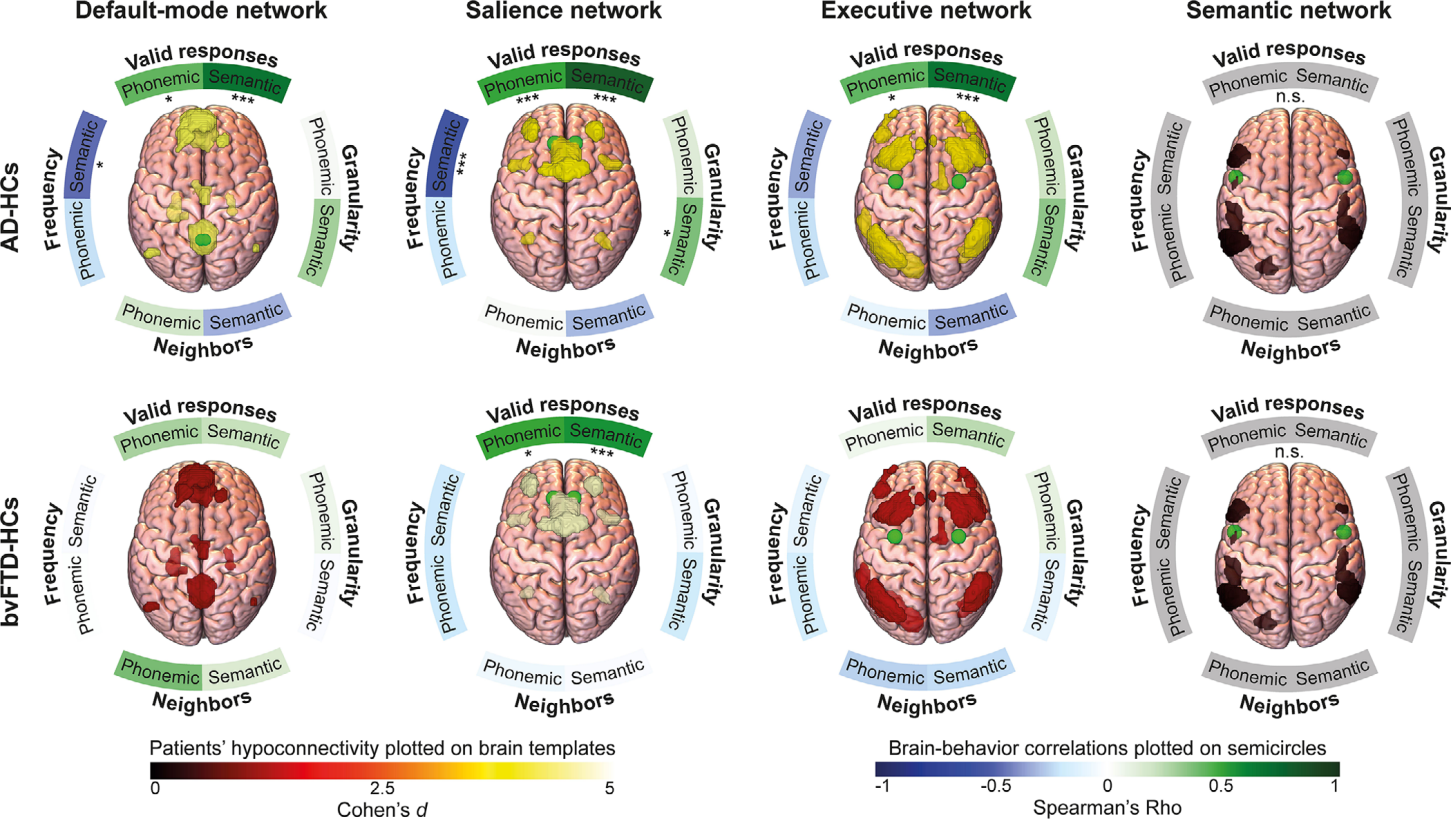
Resting‐state fMRI connectivity results. Brain network masks plotted on brain templates reflect comparison of connectivity between patients and healthy controls in the default‐mode, salience, executive, and semantic network, colored by the effect size of the difference (Cohen's d). Both patient groups presented hypoconnectivity in the default‐mode, salience, and executive networks. Semicircles around brain templates reflects correlations between networks’ connectivity strength and fluency measures, with asterisks indicating the *p*‐value (^***^ *p* < 0.001; ^**^ *p* < 0.01; ^*^ *p* < 0.05) and color indicating correlation strength (Pearson's or Spearman's Rho, as required by data distribution). In the AD‐HC analysis, valid responses in the phonemic and semantic tasks positively correlated with connectivity of the default‐mode, salience, and executive networks. Frequency in the semantic task negatively correlated with connectivity of the default‐mode and salience networks. Granularity in the semantic task positively correlated with connectivity of the salience network. Last, in the bvFTD‐HC analysis, valid responses in the phonemic and semantic tasks positively correlated with connectivity of the salience network. AD, Alzheimer's dementia; bvFTD, behavioral variant frontotemporal dementia; fMRI, functional magnetic resonance imaging; HC, healthy control; n.s., non‐significant difference

In the AD‐HC analysis (Figure 5, top inset), valid responses in the phonemic and semantic tasks positively correlated with connectivity of the default‐mode (phonemic: *r* = 0.39, *P*_FDR_ = 0.04; semantic: *r* = 0.69, *P*_FDR_ < 0.001), salience (phonemic: *r* = 0.49, *P*_FDR_ < 0.001; semantic: *r* = 0.79, *P*_FDR_ < 0.001), and executive (phonemic: *r* = 0.42, *P*_FDR_ = 0.03; semantic: *r* = 0.68, *P*_FDR_ < 0.001) networks. Frequency in the semantic task negatively correlated with connectivity along the default‐mode (*r* = −0.46, *P*_FDR_ = 0.01) and salience (*r* = −0.54, *P*_FDR_ < 0.001) networks. Finally, granularity in the semantic task positively correlated with connectivity of the salience network (*r* = 0.37, *P*_FDR_ = 0.04). No other significant correlations emerged (Supplementary material 15).

In the bvFTD‐HC analysis (Figure 5, bottom inset), valid responses in the phonemic and semantic tasks positively correlated with connectivity of the salience network (phonemic: *r* = 0.49, *P*_FDR_ = 0.01; semantic: *r* = 0.55, *P*_FDR_ < 0.001). No other significant correlations emerged (Supplementary material 15).

#### 3.4.3 EEG connectivity differences and correlations with fluency measures

**FIGURE 6:**
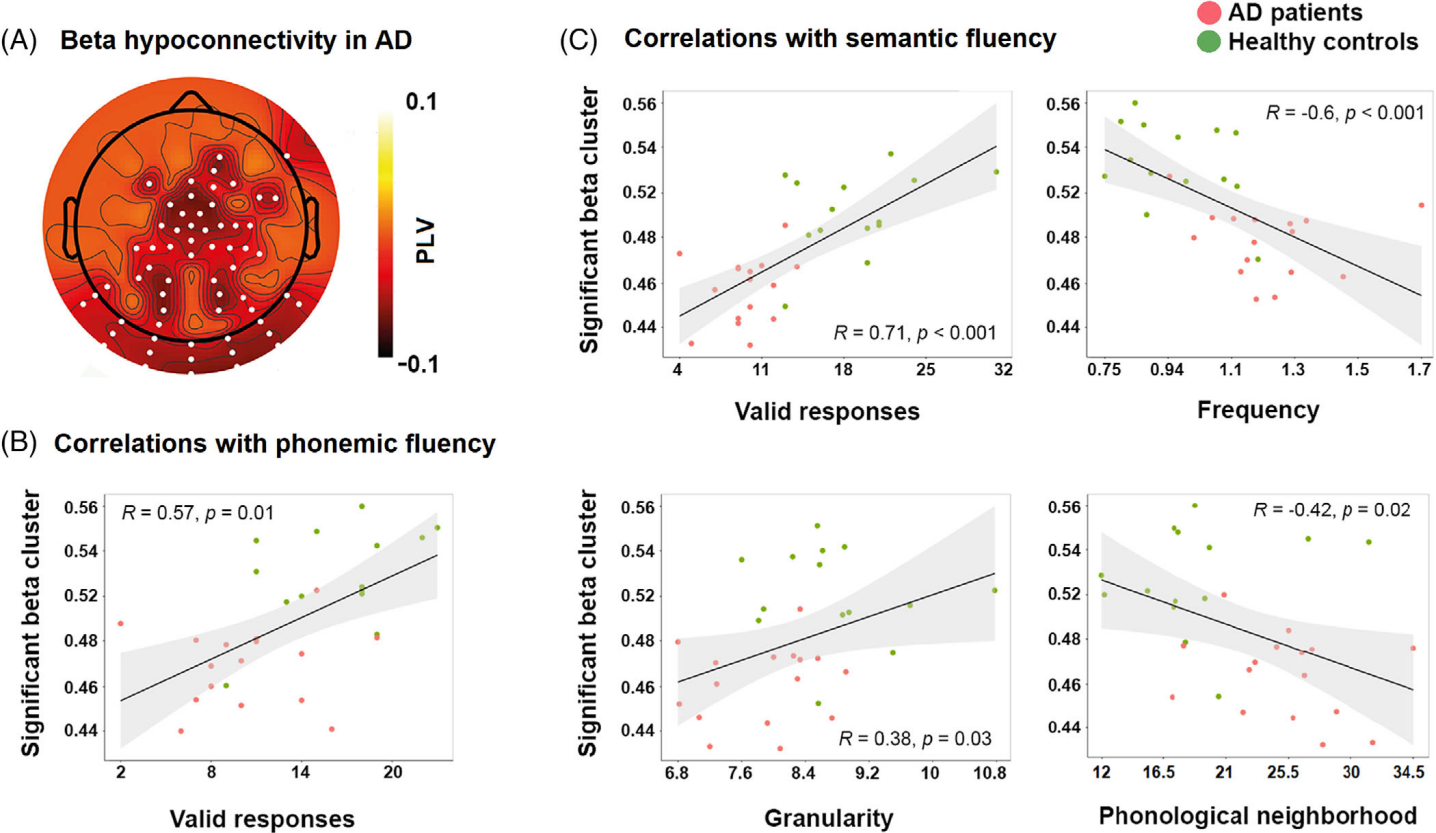
Resting‐state EEG connectivity and correlation results. (A) Topoplot showing phase locking values (PLV) connectivity differences between persons with AD and healthy controls in the beta range (13–30 Hz). (B) Scatterplots showing significant Pearson correlations between mean PLVs in the significant cluster sensors for the semantic fluency measures. (C) Scatterplot showing significant Pearson correlation between mean PLVs in the significant cluster sensors for the phonemic fluency measure. AD, Alzheimer's dementia; EEG, electroencephalography

Relative to controls, persons with AD presented predominantly bilateral occipito‐parieto‐central hypoconnectivity in the beta band (*P*_cluster‐corrected_ = 0.01) (Figure 6A). Conversely, persons with bvFTD showed no significant functional connectivity differences. In the AD‐HC analysis (Figures 6B,C), the beta cluster correlated positively with valid responses in the phonemic (*r* = 0.57, *P*_FDR_ = 0.004) and semantic (*r* = 0.71, *P*_FDR_ < 0.001) tasks, as well as with the responses’ frequency (*r* = − 0.6, *P*_FDR_ < 0.001), granularity (*r* = 0.38, *P*_FDR_ = 0.03), and phonological neighborhood (*r* = − 0.42, *P*_FDR_ = 0.02) in the semantic task; every other correlation for the AD‐HC analysis was not significant (all *P*_FDR_ values > 0.09). No correlations were performed for persons with bvFTD given their null connectivity differences relative to HCs. For full results, see Supplementary material 16.

## 4 DISCUSSION

We aimed to identify discriminatory markers of AD, vis‐à‐vis bvFTD, through multivariate word‐property analysis of verbal fluency. While standard scores (based on valid responses) revealed deficits in both patient groups, the word‐property approach revealed selective alterations for AD in frequency, granularity, and phonological neighborhood. Likewise, word‐property data yielded robust subject‐level classification only for persons with AD, mainly driven by such properties. These, in turn, predicted executive outcomes exclusively in AD. Also, valid responses correlated with widespread atrophy of, and fMRI connectivity among, disease‐sensitive regions in AD and bvFTD, as well as with EEG beta connectivity in AD. Conversely, word‐property features yielded more fine‐grained correlations with atrophy in AD and bvFTD, alongside selective correlations with fMRI and EEG beta connectivity in AD. These findings carry theoretical and clinical implications, as discussed next.

As expected, the standard approach revealed deficits in AD, but also in bvFTD. This pattern corroborates that valid response counts are sensitive but not highly discriminative across neurodegenerative disorders.^8^ Conversely, the word‐property approach revealed selective anomalies for AD. Unlike persons with bvFTD, persons with AD produced significantly more frequent and less granular responses across tasks, alongside words with more phonological neighbors in the semantic task. During word search, then, persons with AD seem to distinctly favor the most accessible spaces of semantic memory, comprised of highly used^1^, ^57^ and conceptually unspecific^16^ items with phonologically common structures.^25^ Indeed, a preference for easily retrievable words has been identified as a cognitive trait that typifies people at risk for AD and that differentiates them from those with other neurodegenerative conditions.^1^, ^16^, ^57^ Thus, word‐property analysis of fluency seems to offer clinical information that escapes the standard approach.

The discriminatory value of word‐property analysis is reinforced by machine learning results. Distributional features of all six word properties yielded low classification of persons with bvFTD vis‐à‐vis HCs. Contrariwise, they afforded robust identification of persons with AD, surpassing outcomes from machine learning analyses of valid responses in the same population.^58^ This result was driven by frequency, granularity, and phonological neighborhood, which yielded good classification even when framed in isolation. Frequency and granularity, indeed, emerged as key discriminatory variables in recent machine learning studies on AD.^16^, ^57^ Accordingly, word‐property analysis, and these variables in particular, are not only sensitive to AD at the group level, but also at the probabilistic subject level.

Correlations with executive outcomes corroborated the selective sensitivity of word‐property features to AD. Whereas valid responses correlated with IFS scores in all groups in both tasks, critical word properties did so almost exclusively for persons with AD. As IFS scores decreased, AD participants favored words with higher frequency, less granularity, and more phonological neighbors. That is, the greater the executive deficits, the greater the reliance on easily retrievable items.^17^, ^25^ These correlations emerged exclusively for the semantic task, reinforcing the relevance of word‐property analysis to tap into canonical dysfunctions of AD, namely, lexico‐semantic processing deficits.^59^ Note that frequency in the semantic task was also associated with IFS scores in bvFTD. This reinforces the link between higher frequency and executive dysfunction, as both groups presented dysexecutive symptoms. Different executive domains could underlie this pattern. For example, inhibitory demands are higher for low than for high‐frequency items,^19^ arguably because retrieval of low‐frequency words requires suppressing competing words that are more consolidated through daily use. While the restricted score ranges of IFS subtests preclude robust correlations with executive sub‐skills, this conjecture could be tested in further dementia studies. Be that as it may, word‐property patterns seem not only useful for discriminating groups and individuals with AD, but also for capturing the severity of their executive symptoms.

Additional insights were provided by correlations with MRI measures. Valid responses were associated with the thickness of disease‐sensitive regions in both the AD‐HC and the bvFTD‐HC analyses. Phonemic fluency was linked to temporal, frontal, and cingulate areas typically compromised in both syndromes,^11^, ^12^, ^13^, ^14^, ^60^ while semantic fluency was associated with temporal regions in the AD‐HC analysis. On the other hand, frequency in the semantic task was the only word property linked to cortical thickness in these two groups. In the AD‐HC analysis, such correlations mainly involved temporo‐parietal and cingulate cortices implicated in semantic memory selection and retrieval.^61^ Conversely, the correlations observed in the bvFTD‐HC analysis predominantly implicated frontotemporal and cingulate cortices, which play a crucial role in executive function and general cognitive outcomes and are frequently impaired in bvFTD.^62^ Note that partly similar substrates have been reported for frequency in previous research,^22^, ^23^ suggesting that both domain‐specific (semantic) and domain‐general (e.g., executive) functions are taxed depending on how common words are. Our results extend such findings, suggesting that this property may capture different neurocognitive patterns in each dementia type.

Interestingly, correlations between word properties and fMRI connectivity did reveal disease‐specific patterns in AD. As in previous works, both patient groups presented hypoconnectivity of the default‐mode, salience, and executive networks.^21^, ^63^, ^64^ Valid responses in the phonemic and semantic tasks correlated with disruptions along the three altered networks in AD and exclusively with the salience network in bvFTD. Crucially, however, word‐property features captured network disruptions only in the case of AD. Specifically, frequency correlated negatively with connectivity along the default‐mode and the salience networks (mirroring previous results in healthy participants^65^), while granularity correlated positively with salience network connectivity. Reduced connectivity of these networks in AD has been associated with cognitive decline^21^ and lexico‐semantic outcomes.^28^ Our results extend such findings by revealing that lower connectivity along such networks disfavors retrieval of less accessible words, as postulated by network accounts of cognitive effort.^66^

Finally, word‐property anomalies were also selectively associated with EEG patterns in persons with AD. This group exhibited reduced bilateral occipito‐parieto‐central connectivity in the beta band as compared to HCs, mirroring previous results.^28^, ^67^ Such hypoconnectivity also differentiated them from persons with bvFTD, who exhibited preserved beta connectivity.^68^ Moreover, across AD patients, beta connectivity correlated with valid responses in both tasks and, more crucially, with frequency, granularity, and phonological neighborhood in the semantic task. In line with previous works,^27^, ^69^ we propose that beta alterations are critically related to word retrievability costs during semantic memory search, offering new neurocognitive insights into AD. More generally, this novel finding reinforces the sensitivity of lexical features to disease‐specific neural disruptions, while informing the thriving agenda of EEG research on neurodegeneration.^51^

Our results bear clinical implications. Verbal fluency tasks are widely used to assess AD, other neurodegenerative disorders,^1^, ^3^, ^8^, ^10^, ^12^, ^13^, ^16^, ^60^ and relevant phenomena, such as cognitive reserve.^70^ Yet, standard scoring diminishes their potential for revealing disease‐differential markers. Conversely, abnormal word‐property features are present in AD but seemingly absent in bvFTD, another form of dementia. Such features enable group‐ and subject‐level identification of AD, while capturing relevant cognitive and multimodal brain signatures. Importantly, our word‐property approach is objective and automated, so that it could be implemented in clinician‐friendly apps offering relevant data capture, processing, and analysis capabilities.^71^, ^72^ In particular, our approach retrieves information from *all* of the participants’ responses, avoiding human decisions on response validity—a challenging issue, given the lack of unified criteria to that end.^15^ Thus, although specific technical skills may be required for its clinical use, our approach may allow repurposing verbal fluency data by targeting the properties rather than the number of responses provided.

Notwithstanding its contributions, our study presents some limitations. First, although the sample size was similar to or larger than those of other studies,^8^, ^16^ replications should be conducted with more participants. Second, this shortcoming prevented us from subdividing each group into phenotypes. Future works might explore the consistency of our findings across amnesic, executive, and behavioral presentations of AD. Third, sociodemographic differences between AD and bvFTD precluded direct between‐group comparisons, which could be pursued with strategically selected samples. Fourth, our protocol lacked measures of socio‐cultural profiles known to modulate neuropsychological performance,^73^ inviting further research targeting relevant domains, such as socioeconomic status. Last, since responses to one condition (e.g., semantic fluency) may be influenced by the instructions of the previous one (e.g., phonemic fluency),^74^ future works could examine word‐property patterns when task order is systematically manipulated.

In sum, unlike standard scoring, word‐property analysis of verbal fluency seems to reveal disease‐differential markers of AD across behavioral and neurocognitive dimensions. The lack of disease‐specificity of fluency outcomes, then, may not be a consequence of the task, but of its canonical analysis approach. Further applications of this framework could inform the global quest for equitable, scalable, discriminatory markers of AD.^75^

## CONFLICT OF INTEREST STATEMENT

None to declare. Author disclosures are available in the supporting information.

## CONSENT STATEMENT

All participants provided written informed consent pursuant to the 1964 Declaration of Helsinki. The study was approved by the Ethics Committees of the involved institutions.

## Supporting information

Supporting information

Supporting information
