# Supplementary material for: Multivariate word properties in fluency tasks reveal markers of Alzheimer's dementia
Source: Alzheimers Dement. 2023 Oct 12;20(2):925–40. doi: 10.1002/alz.13472 (PMC10916979; doi:10.1002/alz.13472)
Supplement: Supplementary file 1 — Supporting information [file ALZ-20-925-s001.docx]

## **Supplementary material**

### Power estimation

To determine the sample size required for our analyses, we ran a power estimation on G*Power 3. Given our statistical design, based on 2x2 mixed effects ANOVAs, we considered three parameters. First, we established an alpha level of *P* = .05. Second, we considered a medium effect size of ƞ_p_^2^ = .06. Finally, we established a power of .90 [1]. This analysis showed that a sample size of 44 per group pair is enough to reach the estimated effects. The group pair with the fewest participants (*n* = 59) reaches a power of 0.97.

**2. Brain atrophy patterns in each patient group**

**Table S1**. Atrophied areas of persons with AD and bvFTD, relative to healthy controls.

| **Group** | **Region** | **Coordinates** | | | **K_E_** | **TFCE** | **Peak *P*_FDR_** |
| --- | --- | --- | --- | --- | --- | --- | --- |
|  |  | ***X*** | ***y*** | ***z*** |  |  |  |
| AD | Right angular gyrus | 43 | -43 | 23 | 26614 | 143575.19 | 0.01 |
|  | Right middle temporal gyrus | 49 | -51 | 16 |  | 137012.83 | 0.01 |
|  | Right middle temporal gyrus | 43 | -20 | -7 |  | 133523.16 | 0.01 |
|  | Left insular cortex | -38 | -19 | -4 | 26432 | 109036.63 | 0.01 |
|  | Left insular cortex | -32 | 4 | 12 |  | 106472.47 | 0.01 |
|  | Left angular gyrus | -39 | -54 | 32 |  | 94199.09 | 0.01 |
| bvFTD | Left orbital medial frontal gyrus | -30 | 48 | -4 | 24222 | 88966.11 | 0.01 |
|  | Left middle frontal gyrus | -21 | 42 | 6 |  | 88141.45 | 0.01 |
|  | Left middle frontal gyrus | -23 | 31 | 22 |  | 81646.62 | 0.01 |
|  | Right triangular inferior frontal gyrus | 37 | 26 | 13 | 23535 | 51751.96 | 0.01 |
|  | Right insular cortex | 31 | -16 | 13 |  | 47643.64 | 0.01 |
|  | Right middle cingulate gyrus | 0 | -14 | 32 |  | 40547.27 | 0.01 |

HCs: healthy controls; AD: Alzheimer’s disease; bvFTD: behavioral variant frontotemporal dementia. TFCE: Threshold free cluster enhancement.

**3. INECO Frontal Screening battery**

The INECO Frontal Screening (IFS) battery [2] is an executive function test validated in Latin American cohorts of persons with dementia [1, 3-6]. It includes eight subtests, namely: (1) motor programming (Luria series, “fist, edge, palm”); (2) conflicting instructions (hitting the table once when the administrator hits it twice, or hitting it twice when the administrator hits it only once); (3) motor inhibitory control; (4) numerical working memory (backward digit span); (5) verbal working memory (months backwards); (6) spatial working memory (modified Corsi tapping test); (7) abstraction capacity (inferring the meaning of proverbs), and (8) verbal inhibitory control (modified Hayling test). The maximum possible score on the IFS is 30 points. As in previous research [1, 3-6], we considered only the global score as an integrative measure of executive performance.

### 4. Comparison of missing values across word properties

**Table S2.** Comparisons of missing values across word properties.

|  | **Groups** | **Comparison** | ***F*** | ***p*-value** | **ƞ_p_^2^** |
| --- | --- | --- | --- | --- | --- |
|  |  |  |  |  |  |
| **Frequency** | AD-HCs | Group  Task  Interaction | 0.17 | .69 | .003 |
|  |  |  | 7.07 | .01 | .11 |
|  |  |  | 2.87 | .10 | .05 |
|  | bvFTD-HCs | Group  Task  Interaction | 0.34  0.20  0.04 | .56 | .01 |
|  |  |  |  | .65 | .004 |
|  |  |  |  | .85 | .001 |
| **Granularity** | AD-HCs | Group  Task  Interaction | 0.63 | .43 | .01 |
|  |  |  | 13.61 | .001 | .19 |
|  |  |  | 1.35 | 25 | .02 |
|  | bvFTD-HCs | Group  Task  Interaction | 0.03  6.96  0.12 | .86 | .001 |
|  |  |  |  | .01 | .11 |
|  |  |  |  | .73 | .002 |
| **Phonological neighborhood** | AD-HCs | Group  Task  Interaction | 0.10  7.48  3.12 | .75  .01  .08 | .002  .12  .05 |
|  | bvFTD-HCs | Group  Task  Interaction | 0.37 | .54 | .01 |
|  |  |  | 0.22 | .64 | 0.004 |
|  |  |  | 0.03 | .86 | .001 |
| **Length** | AD-HCs | Group  Task  Interaction | 0.17 | .69 | .003 |
|  |  |  | 7.07 | .01 | .11 |
|  |  |  | 2.87 | .10 | .05 |
|  | bvFTD-HCs | Group  Task  Interaction | 0.34 | .56 | .01 |
|  |  |  | 0.20 | .65 | 004 |
|  |  |  | 0.04 | .85 | .001 |
| **Familiarity** | AD-HCs | Group  Task  Interaction | 2.80 | .10 | .05 |
|  |  |  | 0.03 | .87 | 001 |
|  |  |  | 0.27 | .61 | .01 |
|  | bvFTD-HCs | Group  Task  Interaction | 0.03 | .87 | < .001 |
|  |  |  | 0.03 | .87 | .001 |
|  |  |  | 0.27 | .61 | .01 |
| **Imageability** | AD-HCs | Group  Task  Interaction | 2.68 | .11 | .05 |
|  |  |  | 0.01 | .01 | < .001 |
|  |  |  | 0.05 | .82 | .001 |
|  | bvFTD-HCs | Group  Task  Interaction | 0.01 | .94 | < .001 |
|  |  |  | 1.26 | .27 | .02 |
|  |  |  | 0.05 | .82 | .001 |
| # Pairwise comparisons are shown only when the interaction effect is significant. HCs: healthy controls; AD: Alzheimer’s disease; bvFTD: behavioral variant frontotemporal dementia. | | | | | |

**5. Additional details about the logistic regression analysis**

We employed a logistic regression model from the Scikit-learn’s linear models library [7], utilizing default hyperparameters, with the exception of the maximum number of iterations for convergence, that was set to 100,000. The model applied an L2 penalty term to the loss function (i.e., Ridge Regression) and had a tolerance of 0.0001 for stopping criteria. The inverse of regularization strength was set to 1.0 (with smaller values indicating stronger regularization). The intercept of the model was also calculated, and the Limited-memory Broyden, Fletcher, Goldfarb, and Shanno (L-BFGS) algorithm was used for optimization [8].

**6. Sociodemographic and cognitive data for the participants with MRI and fMRI recordings**

**Table S3**. Sociodemographic and cognitive data for the participants with MRI and fMRI recordings.

|  | **Persons with AD**  ***N* = 20** | **Persons with bvFTD**  ***N* = 18** | **Healthy**  **controls**  ***N* = 20** | **Statistics**  **(all groups)** | **Pairwise comparisons** | | | |
| --- | --- | --- | --- | --- | --- | --- | --- | --- |
|  |  |  |  |  | **Groups** | | **Estimate** | ***p-*value** |
| **Sociodemographic profiles** | | | | | |  |  |  |
| Sex (F:M) | 13:7 | 9:9 | 13:7 | - | AD-HCs | | 0.00 | 1.00^a^ |
|  |  |  |  |  | bvFTD-HCs | | 0.37 | 0.54^a^ |
| Handedness (L:R) | 3:17 | 0:16 | 1:18 | - | AD-HCs | | 0.23 | 0.64^a^ |
|  |  |  |  |  | bvFTD-HCs | | < 0.001 | 1.00^a^ |
| Years of age | 74.75 (6.00) | 68.39 (8.27) | 70.85 (7.06) | *F* = 3.88  *P* = 0.03^b^ | AD-HCs | | 1.73 | 0.16^c^ |
|  |  |  |  |  | bvFTD-HCs | | -1.06 | 0.47^c^ |
| Years of education | 10.70 (3.91) | 13.71 (5.08) | 13.70 (3.85) | *F* = 3.21  *P* = .05 ^b^ | AD-HCs | | -2.22 | 0.06^c^ |
|  |  |  |  |  | bvFTD-HCs | | 0.004 | 1.00^c^ |
| **Cognitive profiles** | | | | | | | | |
| MoCA | 14.60 (4.82) | 20.29 (6.29) | 26.55 (2.16) | *F* = 34.88  *P* < .001^b^ | AD-HCs | | -8.35 | < .001^c^ |
|  |  |  |  |  | bvFTD-HCs | | -3.97 | < .001^c^ |
| IFS | 13.58 (5.51) | 17.07 (6.03) | 22.13 (2.76) | *F* = 15.75  *P* < .001^b^ | AD-HCs | | -5.59 | < 0.001^c^ |
|  |  |  |  |  | bvFTD-HCs | | -3.00 | 0.01^c^ |
| Data presented as mean (SD), except for sex and handedness. (a) *p*-values calculated via chi-squared test (χ2); (b) p-values calculated via independent measures ANOVA; (c) p-values calculated via Dunnett’s test. AD: Alzheimer’s disease; bvFTD: behavioral variant frontotemporal dementia; HCs: healthy controls; IFS: INECO Frontal Screening; MoCA: Montreal Cognitive Assessment. | | | | | | | | |

**7. MRI and fMRI acquisition parameters in each recruiting center**

**Center 1.** Structural T1 scans were acquired in a 3 T Phillips scanner with a standard head coil and these parameters: matrix dimension = 224 × 224 × 160; 160 slices; voxel size = 1 mm × 1 mm × 1 mm; flip angle = 8°; repetition time (TR) = 8300 ms; echo time (TE) = 3800 ms. Functional spin echo volumes, parallel to the anterior-posterior commissures, covering the whole brain, were sequentially and ascendingly acquired with the following parameters: matrix dimension = 80 × 80 × 49; 49 slices; slice thickness = 3 mm; voxel size in plane = 3 mm × 3 mm × 3 mm; flip angle = 90°; TR = 2640 ms; TE = 30 ms; number of volumes = 220; sequence duration = 10 min.

**Center 2.** Structural T1 scans were acquired in a 3 T Siemens Skyra scanner with a standard head coil and these parameters: matrix dimension = 224 × 224 × 208; 208 slices; voxel size = 1 mm × 1 mm × 1 mm; flip angle = 8°; TR = 1700 ms; TE = 2000 ms. Functional EP2D-BOLD pulse sequences, parallel to the anterior-posterior commissures, covering the whole brain, were acquired sequentially intercalating pair-ascending first with the following parameters: matrix dimension = 76 × 76 × 46; 46 slices; slice thickness = 3 mm; voxel size in plane = 3 mm × 3 mm × 3 mm; flip angle = 90°; TR = 2660 ms; TE = 30 ms; number of volumes = 300; sequence duration = 13.3 min.

**Center 3.** Structural T1 scans were acquired in a 3 T Siemens Skyra scanner with a standard head coil and these parameters: matrix dimension = 256 × 256 × 192; 192 slices; voxel size = 1 mm × 1 mm × 1 mm; flip angle = 8°; TR = 2400 ms; TE = 2000 ms. Functional EP2D-BOLD pulse sequences, parallel to the anterior-posterior commissures, covering the whole brain, were acquired sequentially intercalating pair-ascending first with the following parameters fMRI parameters: matrix dimension = 76 × 76 × 46; 46 slices; slice thickness = 3 mm; voxel size in plane = 3 mm × 3 mm × 3 mm; flip angle = 90°; TR = 2660 ms; TE = 30 ms; number of volumes = 240; sequence duration = 10.5 min.

**8. Movement parameters during the rs-fMRI session**

**Table S4.** Movement parameters and statistics.

| **Movement parameter** | **Persons with AD** | **Persons with bvFTD** | **Healthy controls** | **Statistics** |
| --- | --- | --- | --- | --- |
| Right | 0.09 (0.16) | 0.03 (0.02) | 0.03 (0.01) | *F*(2, 54) = 0.01, *P* = 0.99 |
| Forward | 0.09 (0.05) | 0.08 (0.04) | 0.10 (0.08) | *F*(2, 54) = 0.84, *P* = 0.36 |
| Up | 0.15 (0.10) | 0.11 (0.07) | 0.09 (0.05) | *F*(2, 54) = 1.8, *P* = 0.19 |
| Average translation | 0.11 (0.08) | 0.07 (0.04) | 0.07 (0.04) | *F*(2, 54) = 0.84, *P* = 0.36 |
| Pitch | 0.12 (0.06) | 0.09 (0.06) | 0.07 (0.04) | *F*(2, 54) = 0.07, *P* = 0.80 |
| Roll | 0.06 (0.06) | 0.03 (0.01) | 0.03 (0.01) | *F*(2, 54) = 0.56, *P* = 0.46 |
| Yaw | 0.09 (0.15) | 0.03 (0.02) | 0.03 (0.01) | *F*(2, 54) = 0.04, *P* = 0.84 |
| Average rotation | 0.09 (0.08) | 0.05 (0.03) | 0.04 (0.02) | *F*(2, 54) = 0.15, *P* = 0.70 |

Data presented as mean (*SD*). AD: Alzheimer’s disease; bvFTD: behavioral variant frontotemporal dementia; HCs: healthy controls.

**9. Additional fMRI processing details**

For data processing, we first placed two bilateral seeds on main hubs of each network on cubic regions of interest (ROI) with a size of 7x7x7 voxels [9], located in the following MNI coordinates: (a) default-mode network: posterior cingulate cortex (*x* = 3, *y* = -54, *z* = 27 and *x* = -3, *y* = -54, *z* = 27) [10]; (b) salience network: dorsal anterior cingulate cortex (*x* = 10, *y* = 34, *z* = 24 and *x* = -10, *y* = 34, *z* = 24) [11]; (c) executive network: superior frontal gyri (*x* = 30, *y* = -2, *z* = 62 and *x* = -30, *y* = -2, *z* = 62) [12]; and (d) semantic network: ventral anterior temporal lobe (*x* = -51, *y* = 6, *z* = -39 and *x* = 51, *y* = 6, *z* = -39) [13]. Second, we employed standard masks of each resting-state network [14] to isolate putative brain regions. Third, we averaged the bilateral connectivity values of the seeds within their respective masks, resulting in a weighted symbolic dependence metric (wSDM) connectivity value per network, per subject.

The wSDM [15] captures local and global temporal features of the BOLD signal by weighing a copula-based dependence measure by symbolic similarity. This allows gauging dynamic nonlinear correlations, a crucial aspect of neural connectivity that escapes conventional metrics, such as Pearson’s R. Similar to other statistical copula-based dependency measures [16], wSDM employs rank statistics to bypass the low temporal resolution of fMRI. Here, C is the copula function of the random variables $\left( x,y \right)$ defined on a unit square. According to Sklar’s theorem [17], there exists a unique copula C that links the joint distribution $f$ and the marginals $f_{1}$ , $f_{2}$:

$f\left( x,y \right)=C(f_{1}(x),f_{2}(y))$ (1)

Since the variables $x,y$ are independent if and only if the copula C equals the product copula П defined as the product of their marginal distribution functions [16], the independence of the variables can be measured by a normalized $L^{P}$ distance of C and П:

$\left( h_{p}\iint_{{[0,1]}^{2}} \left| C\left( u^{1},u^{2} \right)-П\left( u^{1},u^{2} \right) \right|du^{1}du^{2} \right)^{\frac{1}{P}}$ , (2)

where 1 ≤ p ≤ ∞ and $h_{p}$ is a normalization constant.

For p = 2, we have Hoeffding’s phi-square (${I\phi}^{2})$ [18],

${{I\phi}^{2}=90\iint_{{[0,1]}^{2}} \left| C\left( u^{1},u^{2} \right)-П\left( u^{1},u^{2} \right) \right|du^{1}du^{2}}$ (3)

where the empirical estimation can be analytically computed [19]. We computed the ${I\phi}^{2}$ with the Information Theoretical Estimators Toolbox [20]. Finally, to account for local variations in the time-series, we represent the increase and decrease of the signal by symbols. This allows comparing sequences of symbols, enabling a dynamical analysis of the dependence between regions. To this end, we defined a symbolic weight sw, being function of the similarity of $\hat{X},\hat{Y}$(i.e., the symbolic transformation of the $x,y$ timeseries), and multiplying the copula-based measure $I\left( x,y \right)$ we obtain the formula for the wSDM:

$wSDM=sw\left( \hat{X},\hat{Y} \right) . I\left( x,y \right)$ (4)

The symbolic weights range from 0 (i.e., minimal similarity) to 1 (i.e., maximal similarity), and were calculated via the Hamming distance [21] between the resulting symbolic strings.

**10. Sociodemographic and cognitive data for the participants with EEG recordings**

**Table S5.** Sociodemographic and cognitive data for the participants with EEG recordings.

|  | **Persons with AD**  *N* = 16 | **Persons with bvFTD**  *N* = 11 | **Healthy**  **controls**  *N* = 14 | **Statistics**  **(all groups)** | **Pairwise comparisons** | | |
| --- | --- | --- | --- | --- | --- | --- | --- |
|  |  |  |  |  | **Groups** | **Estimate** | ***p-*value** |
| **Sociodemographic profiles** | | | | | | | |
| Sex (F:M) | 10:6 | 3:8 | 8:6 | - | AD-HCs | 0.00 | 1.00^a^ |
|  |  |  |  |  | bvFTD-HCs | 1.18 | 0.28g^a^ |
| Handedness (L:R) | 0:15 | 0:11 | 1:13 | - | AD-HCs | 0.001 | 0.97^a^ |
|  |  |  |  |  | bvFTD-HCs | <0.001 | 1.00^a^ |
| Years of age | 74.75 (6.09) | 68.55  (9.91) | 68.57 (7.39) | *F* = 3.16  *P* = .05^b^ | AD-HCs | 2.19 | 0.06^c^ |
|  |  |  |  |  | bvFTD-HCs | -0.01 | 1.00^c^ |
| Years of education | 10.94 (4.17) | 14.18  (5.02) | 13.93 (4.12) | *F* = 2.44  *P* = .10 ^b^ | - | - | - |
|  |  |  |  |  | - | - | - |
| **Cognitive profiles** | | | | | | | |
| MoCA | 16.25 (4.70) | 22.27  (3.93) | 26.57 (2.17) | *F* = 28.06  *P* < .001^b^ | AD-HCs | -7.44 | < .001^c^ |
|  |  |  |  |  | bvFTD-HCs | -2.81 | 0.02^c^ |
| IFS | 15.34 (4.40) | 19.59  (4.42) | 23.21 (2.64) | *F* = 15.34  *P* < .001^b^ | AD-HCs | -5.52 | < .001^c^ |
|  |  |  |  |  | bvFTD-HCs | -2.31 | 0.049^c^ |
| Data presented as mean (SD), except for sex and handedness. (a) p-values calculated via chi-squared test (χ2); (b) p-values calculated via independent measures ANOVA; (c) p-values calculated via Dunnett’s test. AD: Alzheimer’s disease; bvFTD: behavioral variant frontotemporal dementia; HCs: healthy controls; IFS: INECO Frontal Screening; MoCA: Montreal Cognitive Assessment. | | | | | | | |

### 11. Supplementary ANOVA results

**Table S6.** ANOVA results for the standard approach

|  | **Groups** | **Comparison** | ***F*** | ***p-*value** | **ƞp^2^** | **Pairwise comparisons^#^** | | |
| --- | --- | --- | --- | --- | --- | --- | --- | --- |
|  |  |  |  |  |  | **Comparison** | ***p*-value** | **Cohen’s *d*** |
| **Valid responses** | AD-HCs | Group  Task  Interaction | 55.21  4.41  14.68 | < .001  .04  < .001 | .42  .004  .12 | - | - | - |
|  | bvFTD-HCs | Group  Task  Interaction | 22.66  7.78  2.07 | < .001  .006  .15 | .24  .06  .02 | bvFTD, phon – HCs, phon  bvFTD, phon – bvFTD, sem  HCs, phon – HCs, sem  bvFTD, sem – HCs, sem | .03  .85  .04  < .001 | -0.79  -0.21  -0.71  -1.19 |
| # Pairwise comparisons are shown only when the interaction effect is significant. HCs: healthy controls; AD: Alzheimer’s disease; bvFTD: behavioral variant frontotemporal dementia; phon: phonological fluency task; sem: semantic fluency task. | | | | | | | | |

**Table S7.** ANOVA results for the word-property approach.

|  | **Groups** | **Comparison** | ***F*** | ***p*-value** | **ƞp^2^** | **Pairwise comparisons^#^** | | |
| --- | --- | --- | --- | --- | --- | --- | --- | --- |
|  |  |  |  |  |  | **Comparison** | ***p-v*alue** | **Cohen’s *d*** |
| **Frequency** | AD-HCs | Group  Task  Interaction | 9.46  0.05  < 0.01 | .003  .82  .92 | .12  .02  < .001 | - | - | - |
|  | bvFTD-HCs | Group  Task  Interaction | 0.74  2.21  0.01 | .39  .14  .92 | .02  .04  < .001 | - | - | - |
| **Granularity** | AD-HCs | Group  Task  Interaction | 4.63  40.84  1.32 | .03  < .001  .25 | .05  .46  .01 | - | - | - |
|  | bvFTD-HCs | Group  Task  Interaction | 1.41  54.19  0.37 | .24  < .001  .55 | .007  .49  .003 | - | - | - |
| **Phonological neighborhood** | AD-HCs | Group  Task  Interaction | 8.50  12.91  7.66 | .004  > .001  .007 | .02  .05  .06 | AD, phon – HCs, phon  AD, phon – AD, sem  HCs, phon – HCs, sem  AD, sem – HCs, sem | .72  .003  .96  .02 | 0.28  0.79  0.17  0.74 |
|  | bvFTD-HCs | Group  Task  Interaction | 0.72  0.10  0.002 | .40  .76  .96 | .03  .003  > .001 | - | - | - |
| **Length** | AD-HCs | Group  Task  Interaction | 1.94  10.77  1.32 | .17  .001  .25 | .013  .09  .01 | - | - | - |
|  | bvFTD-HCs | Group  Task  Interaction | 0.04  2.05  0.53 | .84  .15  .47 | .02  .01  .005 | - | - | - |
| **Familiarity** | AD-HCs | Group  Task  Interaction | 3.24 | .07  .28  .33 | .03  .05  .009 | - | - | - |
|  |  |  | 1.17 |  |  |  |  |  |
|  |  |  | 0.94 |  |  |  |  |  |
|  | bvFTD-HCs | Group  Task  Interaction | 1.08  5.55  0.54 | .30  .02  .46 | .009  .06  .005 | - | - | - |
| **Imageability** | AD-HCs | Group  Task  Interaction | 0.13  51.64  1.32 | .07  < .001  .25 | .02  .41  .01 | - | - | - |
|  | bvFTD-HCs | Group  Task  Interaction | 0.03  32.80  2.68 | .95  < .001  .10 | .02  .48  .02 | - | - | - |
| # Pairwise comparisons are shown only when the interaction effect is significant. HCs: healthy controls; AD: Alzheimer’s disease; bvFTD: behavioral variant frontotemporal dementia. | | | | | | | | |

### 12. Supplementary ANOVA results after alternative trimming procedures

**Table S8.** ANOVA results for the word-property approach, upon excluding invalid responses.

|  | **Groups** | **Comparison** | ***F*** | ***p*-value** | **ƞp^2^** | **Pairwise comparisons^#^** | | |
| --- | --- | --- | --- | --- | --- | --- | --- | --- |
|  |  |  |  |  |  | **Comparison** | ***p-*value** | **Cohen’s *d*** |
| **Frequency** | AD-HCs | Group | 8.15 | .005 | .13 |  |  |  |
|  |  | Task | 0.94 | .34 | .03 | - | - | - |
|  |  | Interaction | 0.32 | .57 | .003 |  |  |  |
|  | bvFTD-HCs | Group | 1.09 | .30 | .03 |  |  |  |
|  |  | Task | 1.95 | .17 | .03 | - | - | - |
|  |  | Interaction | 0.03 | .86 | < .001 |  |  |  |
| **Granularity** | AD-HCs | Group | 7,23 | .008 | .07 |  |  |  |
|  |  | Task | 42.59 | < .001 | .48 | - | - | - |
|  |  | Interaction | 1.99 | .16 | .02 |  |  |  |
|  | bvFTD-HCs | Group | 2.57 | .11 | .01 |  |  |  |
|  |  | Task | 68.01 | < .001 | .53 | - | - | - |
|  |  | Interaction | 0.94 | .34 | .009 |  |  |  |
| **Phonological neighborhood** | AD-HCs | Group  Task  Interaction | 8.57  9.00  6.03 | .004  .003  .02 | .03  .03  .05 | AD, phon – HCs, phon  AD, phon – AD, sem  HCs, phon – HCs, sem  AD, sem – HCs, sem | .98  .03  .95  .02 | -0.10  -0.63  0.19  0.82 |
|  | bvFTD-HCs | Group | 0.91 | .34 | .03 |  |  |  |
|  |  | Task | 0.18 | .68 | .005 | - | - | - |
|  |  | Interaction | 0.0004 | .99 | < .001 |  |  |  |
| **Length** | AD-HCs | Group | 3,87 | .052 | .02 |  |  |  |
|  |  | Task | 13.98 | < .001 | .11 | - | - | - |
|  |  | Interaction | 2.54 | .11 | .02 |  |  |  |
|  | bvFTD-HCs | Group | 0.12 | .73 | .02 |  |  |  |
|  |  | Task | 1.50 | .22 | .01 | - | - | - |
|  |  | Interaction | 0.43 | .51 | .004 |  |  |  |
| **Familiarity** | AD-HCs | Group | 1.02 | .31 | .02 |  |  |  |
|  |  | Task | 2.79 | .10 | .06 | - | - | - |
|  |  | Interaction | 0.06 | .81 | < .001 |  |  |  |
|  | bvFTD-HCs | Group | 0.16 | .69 | .004 |  |  |  |
|  |  | Task | 3.87 | .052 | .06 | - | - | - |
|  |  | Interaction | 0.06 | .80 | < .001 |  |  |  |
| **Imageability** | AD-HCs | Group | 0.20 | .07 | .009 |  |  |  |
|  |  | Task | 68.82 | < .001 | .46 | - | - | - |
|  |  | Interaction | 3.46 | .07 | .03 |  |  |  |
|  | bvFTD-HCs | Group | 0.03 | .87 | .005 |  |  |  |
|  |  | Task | 31.15 | < .001 | .42 | - | - | - |
|  |  | Interaction | 0.60 | .44 | .006 |  |  |  |
| # Pairwise comparisons are shown only when the interaction effect is significant. HCs: healthy controls; AD: Alzheimer’s disease; bvFTD: behavioral variant frontotemporal dementia; phon: phonological fluency task; sem: semantic fluency task. | | | | | | | | |

**Table S9**. ANOVA results for the word-property approach, upon excluding outliers at 3 SDs.

|  | **Groups** | **Comparison** | ***F*** | ***p*-value** | **ƞp^2^** | **Pairwise comparisons^#^** | | |
| --- | --- | --- | --- | --- | --- | --- | --- | --- |
|  |  |  |  |  |  | **Comparison** | ***p-*value** | **Cohen’s *d*** |
| **Frequency** | AD-HCs | Group  Task  Interaction | 10.11 | .002 | .12 | - | - | - |
|  |  |  | 0.20 | .66 | .003 |  |  |  |
|  |  |  | 1.75 | .19 | .02 |  |  |  |
|  | bvFTD-HCs | Group  Task  Interaction | 0.10  2.65  0.57 | .75 | .01 | - | - | - |
|  |  |  |  | .11 | .09 |  |  |  |
|  |  |  |  | .45 | .007 |  |  |  |
| **Granularity** | AD-HCs | Group  Task  Interaction | 6.38 | .01 | .11 | - | - | - |
|  |  |  | 33.55 | < .001 | .49 |  |  |  |
|  |  |  | 1.37 | .24 | .02 |  |  |  |
|  | bvFTD-HCs | Group  Task  Interaction | 0.37  52.26  0.01 | .54 | < .001 | - | - | - |
|  |  |  |  | < .001 | .53 |  |  |  |
|  |  |  |  | .91 | < .001 |  |  |  |
| **Phonological neighborhood** | AD-HCs | Group  Task  Interaction | 8 8.28  19.26  6.95 | .005  <.001  .01 | .03  .13  .07 | AD, phon – HCs, phon  AD, phon – AD, sem  HCs, phon – HCs, sem  AD, sem – HCs, sem | .72  .003  .96  .02 | -0.28  -0.79  0.17  0.74 |
|  | bvFTD-HCs | Group  Task  Interaction | 0.12 | .73 | .03 | - | - | - |
|  |  |  | 0.32 | .57 | < .001 |  |  |  |
|  |  |  | 0.89 | .35 | .01 |  |  |  |
| **Length** | AD-HCs | Group  Task  Interaction | 1.70 | .20 | .02 | - | - | - |
|  |  |  | 10.21 | .002 | .14 |  |  |  |
|  |  |  | 0.54 | .47 | .006 |  |  |  |
|  | bvFTD-HCs | Group  Task  Interaction | 0.28 | .60 | .01 | - | - | - |
|  |  |  | 1.77 | .19 | .04 |  |  |  |
|  |  |  | 0.002 | .97 | < .001 |  |  |  |
| **Familiarity** | AD-HCs | Group  Task  Interaction | 5.10 | .03 | .03 | - | - | - |
|  |  |  | 0.30 | .58 | .04 |  |  |  |
|  |  |  | 1.87 | .18 | .02 |  |  |  |
|  | bvFTD-HCs | Group  Task  Interaction | 0.53 | .47 | .01 | - | - | - |
|  |  |  | 6.00 | .02 | .12 |  |  |  |
|  |  |  | 0.007 | .93 | < .001 |  |  |  |
| **Imageability** | AD-HCs | Group  Task  Interaction | 0.15 | .70 | .02 | - | - | - |
|  |  |  | 43.42 | < .001 | .46 |  |  |  |
|  |  |  | 0.52 | .47 | .006 |  |  |  |
|  | bvFTD-HCs | Group  Task  Interaction | 0.27 | .61 | < .001 | - | - | -- |
|  |  |  | 38.27 | < .001 | .48 |  |  |  |
|  |  |  | 0.03 | .86 | < .001 |  |  |  |
| # Pairwise comparisons are shown only when the interaction effect is significant. HCs: healthy controls; AD: Alzheimer’s disease; bvFTD: behavioral variant frontotemporal dementia; phon: phonological fluency task; sem: semantic fluency task. | | | | | | | | |

###

### 13. Supplementary results for the multi-feature analyses

**Table S10.** Additional performance measures for the multi-feature analyses, based on logistic regression.

|  | **Group** | **AUC** | **Accuracy** | **Precision** | **Recall** | **F1** | **UAR** |
| --- | --- | --- | --- | --- | --- | --- | --- |
| **All** | AD-HCs | 0.89 (0.09) | 0.84 (0.09) | 0.86 (0.11) | 0.85 (0.14) | 0.85 (0.09) | 0.83 (0.10) |
|  | bvFTD-HCs | 0.62 (0.15) | 0.57 (0.13) | 0.61 (0.16) | 0.59 (0.21) | 0.58 (0.15) | 0.57 (0.13) |
| **Frequency** | AD-HCs | 0.72 (0.14) | 0.66 (0.12) | 0.69 (0.13) | 0.71 (0.18) | 0.68 (0.13) | 0.65 (0.12) |
|  | bvFTD-HCs | 0.60 (0.15) | 0.54 (0.12) | 0.57 (0.13) | 0.65 (0.20) | 0.59 (0.13) | 0.53 (0.12) |
| **Granularity** | AD-HCs | 0.86 (0.10) | 0.76 (0.11) | 0.77 (0.13) | 0.84 (0.15) | 0.79 (0.10) | 0.76 (0.11) |
|  | bvFTD-HCs | 0.63 (0.15) | 0.59 (0.12) | 0.62 (0.13) | 0.69 (0.19) | 0.64 (0.13) | 0.59 (0.12) |
| **Phonological neighborhood** | AD-HCs | 0.82 (0.11) | 0.75 (0.12) | 0.79 (0.13) | 0.77 (0.17) | 0.77 (0.12) | 0.76 (0.12) |
|  | bvFTD-HCs | 0.62 (0.16) | 0.55 (0.12) | 0.60 (0.15) | 0.60 (0.20) | 0.58 (0.14) | 0.55 (0.12) |
| **Length** | AD-HCs | 0.73 (0.14) | 0.66 (0.13) | 0.70 (0.14) | 0.69 (0.18) | 0.68 (0.13) | 0.66 (0.13) |
|  | bvFTD-HCs | 0.55 (0.16) | 0.51 (0.13) | 0.54 (0.13) | 0.62 (0.21) | 0.57 (0.14) | 0.51 (0.13) |
| **Familiarity** | AD-HCs | 0.67 (0.15) | 0.61 (0.13) | 0.63 (0.12) | 0.73 (0.20) | 0.66 (0.13) | 0.60 (0.13) |
|  | bvFTD-HCs | 0.55 (0.16) | 0.50 (0.12) | 0.53 (0.11) | 0.65 (0.20) | 0.58 (0.13) | 0.49 (0.12) |
| **Imageability** | AD-HCs | 0.76 (0.13) | 0.67 (0.12) | 0.70 (0.14) | 0.72 (0.18) | 0.70 (0.12) | 0.67 (0.12) |
|  | bvFTD-HCs | 0.66 (0.15) | 0.62 (0.12) | 0.62 (0.11) | 0.76 (0.18) | 0.68 (0.12) | 0.60 (0.12) |
| **All shuffled** | AD-HCs | 0.49 (0.15) | 0.50 (0.13) | 0.54 (0.13) | 0.60 (0.20) | 0.56 (0.14) | 0.50 (0.13) |
|  | bvFTD-HCs | 0.28 (0.13) | 0.36 (0.12) | 0.40 (0.13) | 0.45 (0.20) | 0.42 (0.15) | 0.36 (0.12) |
| HCs: healthy controls; AD: Alzheimer’s disease; bvFTD: behavioral variant frontotemporal dementia; AUC: area under receiver operating characteristic curve; UAR: unweighted average recall. | | | | | | | |

**Table S11.** Top 15 features in the multivariate classification between persons with AD and HCs.

| **Importance order** | **Tasks** | **Feature** | **Distributional feature** | **Score** |
| --- | --- | --- | --- | --- |
| 1 | Phonemic | Granularity | Kurtosis | 1.02 |
| 2 | Semantic | Neighbors | Kurtosis | 0.70 |
| 3 | Phonemic | Granularity | Maximum | 0.66 |
| 4 | Semantic | Granularity | Maximum | 0.65 |
| 5 | Semantic | Granularity | Skewness | 0.61 |
| 6 | Semantic | Neighbors | Skewness | 0.59 |
| 7 | Phonemic | Neighbors | Kurtosis | 0.57 |
| 8 | Semantic | Neighbors | Median | 0.54 |
| 9 | Semantic | Granularity | Kurtosis | 0.54 |
| 10 | Semantic | Imageability | Skewness | 0.51 |
| 11 | Semantic | Frequency | Mean | 0.50 |
| 12 | Phonemic | Granularity | Skewness | 0.49 |
| 13 | Semantic | Imageability | Kurtosis | 0.47 |
| 14 | Semantic | Frequency | Minimum | 0.47 |
| 15 | Semantic | Neighbors | Mean | 0.44 |
| AD: Alzheimer’s disease; HCs: healthy controls. | | | | |

**14. Correlations between significant fluency variables and whole-brain cortical thickness**

**Table S12.** Correlations between significant fluency variables and whole-brain cortical thickness.

| **Fluency measure** | **Groups** | **Task** | **Region** | **Coordinates** | | | **K_E_** | **TFCE** | **Peak *P*_FDR_** |
| --- | --- | --- | --- | --- | --- | --- | --- | --- | --- |
|  |  |  |  | ***x*** | ***y*** | ***z*** |  |  |  |
| **Valid responses** | AD-HCs | Phonemic | Right rolandic operculum | 50 | -21 | 21 | 21753 | 59664.73 | 0.01 |
|  |  |  | Left putamen | -27 | 12 | 11 | 13615 | 38570.14 | 0.01 |
|  |  |  | Left middle cingulate gyrus | -5 | -26 | 30 | 30 | 6485.08 | < 0.05 |
|  |  |  | Left calcarine fissure | -12 | -55 | 6 | 555 | 5879.06 | 0.04 |
|  |  |  | Left posterior cingulate gyrus | -8 | -44 | 21 | 91 | 5441.3 | 0.04 |
|  |  |  | Left lingual gyrus | -13 | -61 | -8 | 17 | 4011.4 | < 0.05 |
|  |  | Semantic | Right superior temporal gyrus | 40 | -43 | 5 | 24198 | 98991.04 | 0.01 |
|  |  |  | Left middle temporal gyrus | -51 | -56 | 15 | 24363 | 63497.2 | 0.01 |
|  |  |  | Right middle cingulate gyrus | 3 | -15 | 26 | 4 | 2562.17 | < 0.05 |
|  | bvFTD-HCs | Phonemic | Left anterior cingulate gyrus | -16 | 46 | 3 | 16353 | 72304.24 | 0.01 |
|  |  |  | Right middle superior frontal gyrus | 11 | 48 | 8 | 17302 | 64498.18 | 0.01 |
|  |  |  | Left posterior cingulate gyrus | -10 | -48 | 29 | 5 | 8689.43 | < 0.05 |
|  |  |  | Right precuneus | 22 | -45 | 40 | 2 | 8682.67 | < 0.05 |
|  |  |  | Right posterior cingulate gyrus | 7 | -46 | 28 | 4 | 8677.63 | < 0.05 |
|  |  |  | Left precuneus | -9 | -52 | 31 | 16 | 8675.21 | < 0.05 |
|  |  |  | Left precentral gyrus | -27 | -23 | 57 | 4 | 8669.03 | < 0.05 |
|  |  |  | Left middle occipital lobe | -23 | -83 | 13 | 54 | 7981.8 | < 0.05 |
|  |  |  | Left precuneus | -5 | -68 | 32 | 45 | 7890 | < 0.05 |
|  |  |  | Left calcarine fissure | 4 | -87 | 14 | 13 | 5143.73 | < 0.05 |
|  |  |  | Right parahippocampus | 19 | -26 | -11 | 76 | 2661.95 | < 0.05 |
|  |  | Semantic | ----- | --- | --- | --- | --- | --- | --- |
| **Frequency** | AD-HCs | Phonemic | ----- | --- | **---** | --- | --- | --- | --- |
|  |  | Semantic | Left superior temporal pole | 40 | -43 | 5 | 24198 | 98991.04 | 0.01 |
|  |  |  | Right supramarginal gyrus | -51 | -56 | 15 | 24363 | 63497.2 | 0.01 |
|  |  |  | Right middle cingulate gyrus | 3 | -15 | 26 | 4 | 2562.17 | < 0.05 |
|  | bvFTD-HCs | Phonemic | ----- | --- | **---** | --- | --- | --- | --- |
|  |  | Semantic | Rigth middle frontal gyrus | 38 | 9 | 38 | 12340 | 37671.89 | < 0.05 |
|  |  |  | Left middle superior frontal gyrus | -7 | 45 | 26 | 7 | 23499.83 | < 0.05 |
|  |  |  | Left middle frontal gyrus | -29 | 12 | 47 | 10 | 23218.19 | < 0.05 |
|  |  |  | Right orbital middle frontal gyrus | 9 | 43 | -4 | 11 | 21052.79 | < 0.05 |
|  |  |  | Left posterior cingulate gyrus | -9 | -41 | 31 | 1348 | 20341.07 | < 0.05 |
|  |  |  | Left middle cingulate gyrus | -11 | -23 | 35 | 17 | 19931.51 | < 0.05 |
|  |  |  | Right middle temporal gyrus | 58 | -31 | -12 | 57 | 16834.11 | < 0.05 |
|  |  |  | Right middle temporal gyrus | 49 | -10 | -15 | 68 | 16776.65 | < 0.05 |
|  |  |  | Right middle temporal gyrus | 47 | -36 | -2 | 37 | 16433.03 | < 0.05 |
|  |  |  | Right superior temporal gyrus | 56 | -31 | 23 | 99 | 16395.54 | < 0.05 |
|  |  |  | Left cuneus | -9 | -68 | 29 | 150 | 15212.28 | < 0.05 |
|  |  |  | Right supramarginal gyrus | 38 | -34 | 45 | 37 | 14123.3 | < 0.05 |
|  |  |  | Left anterior cingulate gyrus | -1 | 22 | -6 | 4 | 14084.43 | < 0.05 |
|  |  |  | Right amygdala | 29 | 2 | -12 | 93 | 14073.22 | < 0.05 |
|  |  |  | Right parahippocampus | 25 | 10 | -28 | 11 | 14038 | < 0.05 |
|  |  |  | Right putamen | 31 | -20 | 3 | 4 | 14022.83 | < 0.05 |
|  |  |  | Left middle occipital lobe | -43 | -72 | 23 | 26 | 13919.69 | < 0.05 |
|  |  |  | Left supramarginal gyrus | -49 | -25 | 27 | 41 | 13424.86 | < 0.05 |
|  |  |  | Left superior occipital lobe | -18 | -85 | 25 | 178 | 13001.43 | < 0.05 |
|  |  |  | Right parahippocampus | 20 | -7 | -24 | 100 | 12973.87 | < 0.05 |
|  |  |  | Right inferior temporal gyrus | 46 | -57 | -6 | 2 | 12924.38 | < 0.05 |
|  |  |  | Right cuneus | 15 | -80 | 22 | 17 | 11772.89 | < 0.05 |
|  |  |  | Right inferior temporal gyrus | 49 | -26 | -20 | 32 | 9762.06 | < 0.05 |
| **Granularity** | AD-HCs | Phonemic | ----- | --- | **---** | --- | --- | --- | --- |
|  |  | Semantic | ----- | --- | **---** | --- | --- | --- | --- |
|  | bvFTD-HCs | Phonemic | ----- | --- | **---** | --- | --- | --- | --- |
|  |  | Semantic | ----- | --- | **---** | --- | --- | --- | --- |
| **Phonological neighborhood** | AD-HCs | Phonemic | ----- | --- | **---** | --- | --- | --- | --- |
|  |  | Semantic | ----- | --- | **---** | --- | --- | --- | --- |
|  | bvFTD-HCs | Phonemic | ----- | --- | **---** | --- | --- | --- | --- |
|  |  | Semantic | ----- | --- | **---** | --- | --- | --- | --- |
| Non-significant correlations are identified with dotted lines. AD: Alzheimer’s disease; bvFTD: behavioral variant frontotemporal dementia; HCs: healthy controls. TFCE: Threshold free cluster enhancement. | | | | | | | | | |

**15. Correlations between fluency variables and patients’ hypoconnected networks**

**Table S13**. Correlations between significant fluency measures and strength of hypoconnected networks

|  | **Groups** | **Task** | **Brain networks** | | | |
| --- | --- | --- | --- | --- | --- | --- |
|  |  |  | **Default-mode** | **Salience** | **Executive** | **Semantic** |
| **Valid responses** | AD-HCs | Phonemic | *r =* 0.39, *p =* 0.04 | *r =* 0.49 *p <* 0.001 | *r =* 0.42 *p =* 0.03 | ----- |
|  |  | Semantic | *r =* 0.69 *p <* 0.001 | *r =* 0.79 *p <* 0.001 | *r =* 0.68 *p <* 0.001 |  |
|  | bvFTD-HCs | Phonemic | *r =* 0.28 *p =* 0.39 | *r =* 0.49 *p =* 0.01 | *r =* 0.06 *p =* 0.78 | ----- |
|  |  | Semantic | *r =* 0.21 *p =* 0.55 | *r =* 0.55 p < 0.001 | *r =* 0.24 *p =* 0.47 |  |
| **Frequency** | AD-HCs | Phonemic | *r =* -0.22 *p =* 0.23 | *r =* -0.20 *p =* 0.31 | *r =* -0.22 *p =* 0.25 | ----- |
|  |  | Semantic | *r =* -0.46 *p =* 0.01 | *r =* -0.54 p < 0.001 | *r =* -0.34 *p =* 0.06 |  |
|  | bvFTD-HCs | Phonemic | *r =* 0.01 *p =* 0.98 | *r =* -0.20 *p =* 0.50 | *r =* -0.18 *p =* 0.57 | ----- |
|  |  | Semantic | *r =* -0.03 *p =* 0.98 | *r =* -0.19 *p =* 0.50 | *r =* -0.10 *p =* 0.76 |  |
| **Granularity** | AD-HCs | Phonemic | *r =* 0.04 *p =* 0.80 | *r =* 0.13 *p =* 0.47 | *r =* 0.20 *p =* 0.25 | ----- |
|  |  | Semantic | *r =* 0.29 *p =* 0.11 | *r =* 0.37 *p =* 0.04 | *r =* 0.33 *p =* 0.06 |  |
|  | bvFTD-HCs | Phonemic | *r =* 0.07 *p =* 0.98 | *r =* -0.05 *p =* 0.97 | *r =* 0.10 *p =* 0.76 | ----- |
|  |  | Semantic | *r =* -0.03 *p =* 0.98 | *r =* -0.19 *p =* 0.50 | *r =* -0.10 *p =* 0.76 |  |
| **Phonological neighborhood** | AD-HCs | Phonemic | *r =* 0.18 *p =* 0.30 | *r =* 0.03 *p =* 0.87 | *r =* -0.08 *p =* 0.62 | ----- |
|  |  | Semantic | *r =* -0.32 *p =* 0.09 | *r =* -0.30 *p =* 0.11 | *r =* -0.34 *p =* 0.06 |  |
|  | bvFTD-HCs | Phonemic | *r =* 0.38 *p =* 0.17 | *r =* -0.06 *p =* 0.97 | *r =* -0.25 *p =* 0.47 | ----- |
|  |  | Semantic | *r =* 0.15 *p =* 0.77 | *r =* -0.03 *p =* 0.97 | *r =* -0.23 *p =* 0.47 |  |

**16. Correlations between significant fluency measures and beta connectivity clusters**

**Table S14.** Pearson correlations between significant fluency measures and beta connectivity clusters.

|  | **Groups** | **Task** | ***p-v*alue**  **(FDR-corrected)** | **Pearson’s *r*** |
| --- | --- | --- | --- | --- |
| **Valid responses** | AD-HCs | Phonemic | .01 | 0.57 |
|  |  | Semantic | < .001 | 0.71 |
| **Frequency** | AD-HCs | Phonemic | .09 | -0.37 |
|  |  | Semantic | < .001 | -0.60 |
| **Granularity** | AD-HCs | Phonemic | .85 | -0.09 |
|  |  | Semantic | .04 | 0.38 |
| **Phonological neighborhood** | AD-HCs | Phonemic | .87 | 0.03 |
|  |  | Semantic | .03 | -0.42 |
| Pearson’s r and FDR-corrected *p*-values are provided for correlations in AD-HCs. Note that no correlations were examined for persons with behavioral variant frontotemporal dementia given that they exhibited no significant functional connectivity alterations. AD: Alzheimer’s disease; HCs: healthy controls. | | | | |

**Supplementary references**

[1] Birba A, Fittipaldi S, Cediel Escobar JC, Gonzalez Campo C, Legaz A, Galiani A, et al. Multimodal neurocognitive markers of naturalistic discourse typify diverse neurodegenerative diseases. Cereb Cortex. 2022;32:3377-91.

[2] Torralva T, Roca M, Gleichgerrcht E, Lopez P, Manes F. INECO Frontal Screening (IFS): a brief, sensitive, and specific tool to assess executive functions in dementia. J Int Neuropsychol Soc. 2009;15:777-86.

[3] Legaz A, Prado P, Moguilner S, Báez S, Santamaría-García H, Birba A, et al. Social and non-social working memory in neurodegeneration. Neurobiol Dis. 2023;183:106171.

[4] Maito MA, Santamaría-García H, Moguilner S, Possin KL, Godoy ME, Avila-Funes JA, et al. Classification of Alzheimer's disease and frontotemporal dementia using routine clinical and cognitive measures across multicentric underrepresented samples: A cross sectional observational study. Lancet Reg Health Am. 2023;17.

[5] Legaz A, Abrevaya S, Dottori M, Gonzalez Campo C, Birba A, Martorell Caro M, et al. Multimodal mechanisms of human socially reinforced learning across neurodegenerative diseases. Brain. 2022;145:1052-68.

[6] Sanz C, Carrillo F, Slachevsky A, Forno G, Gorno Tempini ML, Villagra R, et al. Automated text-level semantic markers of Alzheimer's disease. Alzheimer's Dement: Diagn Assess Dis Monit. 2022;14:e12276.

[7] Pedregosa F, Varoquaux G, Gramfort A, Michel V, Thirion B, Grisel O, et al. Scikit-learn: Machine learning in Python. J Mach Learn Res. 2011;12:2825-30.

[8] Byrd RH, Lu P, Nocedal J, Zhu C. A Limited memory algorithm for bound constrained optimization. SIAM J Sci Comp. 1995;16:1190-208.

[9] Koslov K, Mendes WB, Pajtas PE, Pizzagalli DA. Asymmetry in resting intracortical activity as a buffer to social threat. Psychol Sci. 2011;22:641-9.

[10] Uddin LQ, Kelly AM, Biswal BB, Castellanos FX, Milham MP. Functional connectivity of default mode network components: correlation, anticorrelation, and causality. Hum Brain Mapp. 2009;30:625-37.

[11] Seeley WW, Menon V, Schatzberg AF, Keller J, Glover GH, Kenna H, et al. Dissociable intrinsic connectivity networks for salience processing and executive control. J Neurosci. 2007;27:2349-56.

[12] Boord P, Madhyastha TM, Askren MK, Grabowski TJ. Executive attention networks show altered relationship with default mode network in PD. NeuroImage Clin. 2017;13:1-8.

[13] García AM, Moguilner S, Torquati K, García-Marco E, Herrera E, Muñoz E, et al. How meaning unfolds in neural time: Embodied reactivations can precede multimodal semantic effects during language processing. NeuroImage. 2019;197:439-49.

[14] Shirer WR, Ryali S, Rykhlevskaia E, Menon V, Greicius MD. Decoding subject-driven cognitive states with whole-brain connectivity patterns. Cereb Cortex. 2012;22:158-65.

[15] Moguilner S, García AM, Mikulan E, Hesse E, García-Cordero I, Melloni M, et al. Weighted Symbolic Dependence Metric (wSDM) for fMRI resting-state connectivity: A multicentric validation for frontotemporal dementia. Sci Rep. 2018;8:11181.

[16] Nelsen R. An Introduction to Copulas. New York: Springer Series in Statistics; 2006.

[17] Sklar A. Fonctions de repartition ´ a` n dimensions et leurs marges. Publ Inst Statist Univ Paris. 1959;8.

[18] Hoeffding W. Masstabinvariante Korrelationstheorie. Schrift Math Seminars Inst Angew Math Univ Berlin. 1940;5:181-233.

[19] Gaißer S, Ruppert M, Schmid F. A multivariate version of Hoeffding’s Phi-Square. J Multivar Anal. 2010;101:2571-86.

[20] Szabo Z. Information Theoretical Estimators Toolbox. J Mach Learn Res. 2014;15.

[21] Lesk A. Introduction to Bioinformatics. Oxford: Oxford University Press; 2002.
